# Worldwide Occurrence and Impact of Human Trichinellosis, 1986–2009

**DOI:** 10.3201/eid1712.110896

**Published:** 2011-12

**Authors:** K. Darwin Murrell, Edoardo Pozio

**Affiliations:** Author affiliations: University of Copenhagen, Copenhagen, Denmark (K.D. Murrell);; Istituto Superiore di Sanità, Rome, Italy (E. Pozio)

**Keywords:** trichinellosis, Trichinella, morbidity, mortality, incidence, epidemiology, gender, pork, zoonoses, parasitic, trichinosis, CME

## MEDSCAPE CME

Medscape, LLC is pleased to provide online continuing medical education (CME) for this journal article, allowing clinicians the opportunity to earn CME credit.

This activity has been planned and implemented in accordance with the Essential Areas and policies of the Accreditation Council for Continuing Medical Education through the joint sponsorship of Medscape, LLC and Emerging Infectious Diseases. Medscape, LLC is accredited by the ACCME to provide continuing medical education for physicians.

Medscape, LLC designates this Journal-based CME activity for a maximum of 1 *AMA PRA Category 1 Credit(s)*^TM^. Physicians should claim only the credit commensurate with the extent of their participation in the activity.

All other clinicians completing this activity will be issued a certificate of participation. To participate in this journal CME activity: (1) review the learning objectives and author disclosures; (2) study the education content; (3) take the post-test with a 70% minimum passing score and complete the evaluation at **www.medscape.org/journal/eid**; (4) view/print certificate.

**Release date: November 23, 2011; Expiration date: November 23, 2012**

## Learning Objectives

Upon completion of this activity, participants will be able to:

- Evaluate epidemiologic patterns of trichinellosis
- Analyze the clinical presentation of trichinellosis
- Distinguish the most common animal source of trichinellosis

## MEDSCAPE CME Editor

**Caran R. Wilbanks,** Technical Writer/Editor, *Emerging Infectious Diseases. Disclosure: Caran R. Wilbanks has disclosed the following relevant financial relationship: partner is employed by McKesson Corporation.*

## MEDSCAPE CME AUTHOR

**Charles P. Vega, MD**, Associate Professor; Residency Director, Department of Family Medicine, University of California, Irvine. *Disclosure: Charles P. Vega, MD, has disclosed no relevant financial relationships.*

## AUTHORS

*Disclosures:* ***K. Darwin Murrell, PhD;*** *and* ***Edoardo Pozio, PhD,*** *have disclosed no relevant financial relationships.*
