# Supplementary material for: Worldwide Occurrence and Impact of Human Trichinellosis, 1986–2009
Source: Emerg Infect Dis. 2011 Dec;17(12):2194–202. doi: 10.3201/eid1712.110896 (PMC3311199; doi:10.3201/eid1712.110896)
Supplement: Technical Appendix — Additiona information on Trichinellosis infections and resources used in this study. [file 11-0896-Techapp_43p.pdf]

Article DOI: http://dx.doi.org/10.3201/eid1712.110896

Worldwide Occurrence and Impact of Human Trichinellosis, 1986–2009

Technical Appendix

Section A. Total cases and incidence of human *Trichinella* infections by country and World Health Organization region, 1986–2009*

Region/Country	Period	No. cases	Average incidence (range/dates)†	References
African Region		28		
Ethiopia	1986<br>1990	8<br>20	0.02<br>0.04	Kefenie et al., 1988<br>Kefenie and Bero, 1992
Region of the Americas		7,179		
Canada	1987–2009	257	21.3 for Kewatin region (1989–1995)	Heinzig, 1996<br>Schellenberg et al., 2003<br>Ancelle et al., 2006<br>Gaulin et al., 2006<br>Houzé et al., 2009<br>Serhir et al., 2001<br>Møller et al., 2005<br>McIntyre et al., 2007
USA	1987–2007	652	0.016<br>(0.01–0.04/1987–1990)<br>0.014<br>(0.001–0.03/1991–1996)<br>0.005<br>(0.004–0.005/1997–2001)<br>0.004<br>(0.001–0.002/2002–2007)	McAuley et al., 1991<br>Moorhead et al., 1999<br>Roy et al., 2003<br>Kennedy et al., 2009<br>Smith et al. 2004<br>Nelson et al., 2003<br>Madariaga et al., 2007<br>Centers for Disease Control and Prevention, 1987<br>Landry et al., 1992<br>Petri et al., 1988<br>Graves et al., 1996<br>McAuley et al., 1992<br>Dworkin et al., 1996
Mexico	1986–2001	351	0.25 for State of Mexico (1986–1997)	Ortega-Pierres et al., 2000<br>Chapa-Ruiz et al., 2001
Argentina	1990–2005	5,221	1.48 (1990–1999)	Bolpe and Boffi, 2001<br>Nunez et al., 2008<br>Rossi et al., 2007
Chile	1991–2004	698	0.36 (1996–2000)<br>0.2 (2000)	Schenone et al., 2002<br>Morales et al., 1999<br>Garciá et al., 2005
Eastern Mediterranean Region		50		
Iran	2007	6	0.008	Kia et al., 2008
Lebanon	1995	44	1.25	Haim et al., 1997
European Region		56,912		
Belarus	1986–1989	16	0.08<br>0.55	Ivanov et al., 1990<br>Skripova and Kovchur, 1994
Bosnia-Herzegovina	1993–2003	1,600	4.1 (0.1–8.0)	Cuperlovic et al., 2005
Bulgaria	1990–2006	4,108	2.9 (0.3–7.4/1990–1999)<br>2.4 (0.7–4.0/2000–2006)	Kurdova-Mintcheva et al., 2009<br>Petkova et al., 2008

Region/Country	Period	No. cases	Average incidence (range/dates)†	References
Croatia	1994–1999	1,350	4.8 (2.1–12.3)	Aleraj, 2008
	2000–2009	760	1.7 (0.02–3.9)	Croation National Institute of Public Health, 2010
				Marinculic et al., 2001
				Vojnikovic et al., 2001
				Puljiz et al., 2005
				Cvitovic et al., 2007
				Venus et al., 2008
Czech Republic	1986–2009	31	0.01	National Reference Laboratory for Tissue Helminthosis, 2010
Estonia	1986–2009	91	0.2 (0.0–2.9)	Epshtein, 2010
France	1986–1989	11	0.005 (0.0–0.02)	Dupouy-Camet et al., 2010
	1990–1999	1,158	0.19 (0.0–0.95)	Durant et al., 1991
	2000–2009	34	0.006 (0.0–0.02)	Roumier et al., 1992
				Soulé, 1992
				Ancelle et al., 1994
				Gari-Toussaint et al., 1994
				Soulé, 1994
				Basset et al., 1995
				Bernard et al., 1995
				Laurichesse et al., 1997
				Maillot et al., 1997
				Ancelle et al., 1998
				Dupouy-Camet et al., 1998
				Haeghebaert et al., 1998
				Hemery and Haeghebaert, 1999
				Ranque et al., 2000
				Gari-Toussaint et al., 2004
				Gari-Toussaint et al., 2005
				Ancelle et al., 2006
				De Bruyne et al., 2006
				Gaillard et al., 2007
Georgia	1988	3	0.05	Ivanov et al., 1990
Germany	1986–1989	19	0.005 (0.003–0.007)	Anonymous, 2010
	1990–1999	105	0.009 (0.0–0.01)	Robert Koch Institute, 2010
	2000–2009	61	0.005 (0.0–0.01)	Nothdurft et al., 1995
				Littmann et al., 2006
				Nockler et al., 2007
				Jansen et al., 2008
Greece	2009	1	0.008	Boutsini, 2009
Hungary	1986–1989	74	0.18 (0.08–0.32)	Glatz et al., 2010
	1990–1999	27	0.027	
	2000–2009	57	0.057 (0.0–0.13)	
Ireland	2007	2	0.04	McHugh et al., 2007
Israel	2002	30	0.5	Hefer et al., 2004
	2004	200	3.0	Marva et al., 2005
Italy	1986–1989	448	0.19 (0.0–0.7)	E. Pozio, unpub. data
	1990–1999	649	0.01 (0.0–0.9)	Pozio et al., 1986
	2000–2009	84	0.01 (0.0–0.07)	Pozio et al., 1987
				Di Bari et al., 1990
				Frongillo et al., 1992
				Pozio et al., 1993
				Pagni et al., 1994
				Pozio et al., 1998
				Pozio et al., 2001
				Pozio and Marucci, 2003
				Pozio et al., 2006
				Pozio et al., 2009
Kyrgyzstan	1996	10	0.2	Witschi, 2010
Latvia	1986–1989	22	0.20 (0.07–0.34)	NRL of Latvia Dr. G. Deksne
	1990–1999	345	1.37 (0.60–3.40)	
	2000–2009	269	1.13 (0.20–3.80)	
Lithuania	1990–2004	3,705	6.6 (0.6–21.8)	Bartuliene et al., 2005
	1989	75	2.0	Rockiene, 1996
	2005–2009	199	1.2 (0.4–3.4)	EpiNorth, 2010
				Malakauskas, 2002
				Bartuliene et al., 2009
Macedonia	1992	6	0.3	Hristovski et al., 1992

Region/Country	Period	No. cases	Average incidence (range/dates)†	References
Poland	1986–1991	955	0.4 (0.05–0.8)	Adonajlo, 1988–1993
	1992	219	0.6	Koncki, 1994
	1993–1999	1,085	0.4 (0.05–1.5)	Seroka, 1995–2001
	2000–2001	88	0.1	Przybylska, 2002–2003
	2002–2007	737	0.3 (0.1–0.7)	Sadkowska-Todys and Gołab, 2005–2009
Romania	1986–1989	2,621	2.9 (1.7–3.9)	Neghina et al., 2010a
	1990–1999	20,059	8.57 (4.1–16.1)	Blaga et al., 2007
	2000–2007	5,884	3.3 (1.8–5.9)	Neghina et al., 2009a
				Neghina et al., 2009b
				Nemet et al., 2009
				Neghina et al., 2010b
Russia	1996–2002	971	0.48 (0.3–0.6)	Ozeretskovskaya et al., 2005
Serbia	1994–2003	5,210	5.05 (1.8–7.8)	Cuperlovic et al., 2005
				Cuperlovic et al., 2001
				Djordjevic et al., 2003
Slovakia	1986–1992	83	0.22 (0.0–0.64)	Dubinský et al., 1993
	1998	336	6.2	Dubinský et al., 2001
	2001	11	0.2	Reiterova et al., 2007
	2008	10	0.2	Paraličová et al., 2009
Slovenia	1989	200	10.5	Brglez, 1996
	1995	2	0.1	Saletinger et al., 2007
	2006	1	0.05	
Spain	1986–1989	233	0.15 (0.0–0.3)	Anonymous, 2010
	1990–1999	607	0.13 (0.02–0.40)	Anonymous, 1986
	2000–2009	404	0.08 (0.0–0.25)	Anonymous, 1987
				Benitez et al., 1987
				Anonymous, 1988
				de la Torre et al., 1989
				Serrano et al., 1989
				Anonymous, 1990
				Cobo et al., 1991
				Mangas Gallardo and Tello Anchuela, 1994
				de la Cruz de Julián et al., 1994
				Anonymous, 1995
				Tiberio et al., 1995
				Tiberio et al., 1997
				Rodriguez-Osorio et al., 1999
				Martinez Corral et al., 2000
				Lopez Hernandez et al., 2001
				Cortés Blanco et al., 2002
				Gomez Garcia et al., 2003
				Herraez Garcia et al., 2003
				Gallardo et al., 2007
				Arevalo et al., 2009
Switzerland	1994	3	0.04	Lupinc et al., 2003
				J.C. Lozano-Becerra, pers. comm.
Turkey	2003	7	0.01	Heper et al., 2005
	2004	418	0.59	Akkoc et al., 2009
United Kingdom	1999	7	0.01	Milne et al., 2001
Ukraine	1986–1989	147	0.06 (0.04–0.10)	Ukraine Ministry of Health, 2010
	1990–1999	955	0.18 (0.09–0.3)	
	2000–2009	108	0.018 (0.0–0.08)	
South-East Asia Region		219		
Thailand	1993–2007	216	0.35	Limsuwan and Siriprasert 1994
				Charkrit, 1998
				Jongwutiwes et al., 1998
				Watt et al., 2000
				Chotmongkul et al., 2005
				Khumjui et al., 2008
India	1996–2002	3	0.0003	Alipuria et al., 1996
				Handa et al., 2000
				Mohan et al., 2002
Western Pacific Region		1,344		

Region/Country	Period	No. cases	Average incidence (range/dates)†	References
People's Republic of China	1995–2009	1,137	Yunan, 1.0<br>Henan, 0.001<br>Tibet, 1.74<br>Sichuan, 0.028<br>Hubei, 0.03<br>(2000–2003)	Cui et al., 1997<br>Lo et al., 2009<br>Wang and Cui, 2001<br>Xu et al., 1995<br>Gong et al., 2008<br>Ye et al., 2003<br>Ci et al., 2003
Japan	1999–2005	4	NA	Kusuhara et al., 1999<br>Shiota et al., 1999<br>Nakamura et al., 2003<br>Yoshikawa et al., 2005
South Korea	1999–2003	8	0.016	Sohn et al., 2003<br>Kim et al., 2003<br>Lee et al., 1999
Laos	2004–2006	123	2.09	Sayasone et al., 2006<br>Taybouavone et al., 2009<br>Suwansrinen et al., 2007<br>Barennes et al., 2008
Singapore	1998	25	0.64	Kurup et al., 2000
Vietnam	2001–2004	47	0.058	Taylor et al., 2009<br>De et al., 2006

*Human trichinellosis infections acquired in countries different from those where the disease was developed and diagnosed were not included in this table. NA, insufficient data for incidence calculation.
†Incidence/10^5 person-years. The incidence period is reported because the data may have been obtained over a period briefer than the interval on which total cases are based. For some countries, incidence was not reported and was calculated from data available in the report referenced.

References

African Region

Ethiopia

Kefenie H, Bero G. Trichinosis from wild boar meat in Gojjam, north-west Ethiopia. Trop Geogr Med. 1992;44:278–80. PubMed

Kefenie H, Wolde H, Abuherpo M. Trichinosis from wild boar meat in Arsi, central Ethiopia. Ethiop Med J. 1988;26:97–100. PubMed

Region of the Americas

Canada

Ancelle T, Bruyne A, Nuang M, Poisson DM, Prazuck T, Fur A, et al. Epidemic of *Trichinella nativa* trichinosis caused by consumption of bear meat, France, 2005. Bulletin Épidémiologique Hebdomadaire. 2006;14:96–8.

Public Health Agency of Canada. Outbreak of trichinellosis in French hunters who ate Canadian black bear meat. Can Commun Dis Rep. 2006;32:109–12. PubMed

Heinzig L. Case reviews—botulism and trichinosis. EpiNorth. 1996;8(4):7.

Houzé S, Ancelle T, Matra R, Boceno C, Carlier Y, Gajadhar AA, et al. Trichinellosis acquired in Nunavut, Canada in September 2009: meat from grizzly bear suspected. Euro Surveill. 2009;14:5. PubMed

McIntyre L, Pollack SL, Fyfe M, Gajadhar A, Issac-Renton J, Fung J, et al. Trichinosis from consumption of wild game meat. Can Med Assoc J. 2007;176:449–51. doi:10.1503/cmaj.061530

Møller LN, Petersen E, Kapel CMO, Melbye M, Koch A. Outbreak of trichinellosis associated with consumption of game meat in West Greenland. Vet Parasitol. 2005;132:131–6. PubMed doi:10.1016/j.vetpar.2005.05.041

Schellenberg RS, Tan B, Irvine JD, Stockdale DR, Gajadhar AA, Serhir B, et al. An outbreak of trichinellosis due to consumption of bear meat infected with *Trichinella nativa* in 2 northern Saskatchewan communities. J Infect Dis. 2003;188:835–43. PubMed doi:10.1086/378094

Serhir B, MacLean JD, Healey S, Forbes L. Outbreak of trichinellosis associated with arctic walruses in northern Canada, 1999. Can Commun Dis Rep. 2001;27–04:31–36.

United States

Centers for Disease Control and Prevention. Trichinosis—Hawaii. MMWR Morb Mortal Wkly Rep. 1987;36:14–6. PubMed

Dworkin MS, Gamble HR, Zarlenga DS, Tennican PO. Outbreak of trichinellosis associated with eating cougar jerky. J Infect Dis. 1996;174:663–6. PubMed doi:10.1093/infdis/174.3.663

Graves T, Harkness J, Crutcher JM. Case report: locally aquired trichinosis in an immigrant from Southeast Asia. J Okla State Med Assoc. 1996;89:402–4. PubMed

Kennedy ED, Hall RL, Montgomery SP, Pyburn DG, Jones JJ. Trichinellosis surveillance—United States, 2002–2007. MMWR Surveill Summ. 2009;58(9):1–7. PubMed

Landry SM, Kiser D, Overby T, Mays B, Caplen CW. Trichinosis: common source outbreak related to commercial pork. South Med J. 1992;85:428–9. PubMed doi:10.1097/00007611-199204000-00022

Madariaga MG, Cachay ER, Zarlenga DS. Case report: a probable case of human neurotrichinellosis in the United States. Am J Trop Med Hyg. 2007;77:347–9. PubMed

McAuley JB, Michelson MK, Schantz PM. Trichinellosis surveillance, United States, 1987–90. MMWR Surveill Summ. 1991;40(SS-3):35–42. PubMed

McAuley JB, Michelson MK, Hightower AW, Engeran S, Wintermeyer LA, Schantz PM. A trichinosis outbreak among Southeast Asian refugees. Am J Epidemiol. 1992;135:1404–10. PubMed

Moorhead A, Grunenwald PE, Dietz VJ, Schantz PM. Trichinellosis in the United States, 1991–1996: declining but not gone. Am J Trop Med Hyg. 1999;60:66–9. PubMed

Nelson M, Wright TL, Pierce A, Krogwold RA. A common source outbreak of trichinosis from consumption of bear meat. J Environ Health. 2003;65:16–19, 24. PubMed

Petri WA, Holsinger JR, Pearson RD. Common-source outbreak of trichinosis associated with eating raw home-butchered pork. South Med J. 1988;81:1056–8. PubMed doi:10.1097/00007611-198808000-00029

Roy SL, Lopez AS, Schantz PM. Trichinellosis surveillance—United States, 1997–2001. MMWR Surveill Summ. 2003;52(6):1–8. PubMed

Centers for Disease Control and Prevention. Trichinellosis associated with bear meat—New York and Tennessee, 2003. MMWR Morb Mortal Wkly Rep. 2004;53:606–10. PubMed

Mexico

Chapa-Ruiz MR, González-Pantaleón D, Morales-Galán A, Contreras-Ramos A, Salinas-Tobón MR, Martínez Y, et al. A follow-up study of the human class and subclass antibody response developed against the adult stage of *Trichinella spiralis.* Parasite. 2001;8(Suppl):S163–7. PubMed

Ortega-Pierres MG, Arriga C, Yepez-Mulia L. Epidemiology of trichinellosis in Mexico, Central and South America. Vet Parasitol. 2000;93:201–25. PubMed doi:10.1016/S0304-4017(00)00342-3

Argentina

Bolpe J, Boffi R. Human trichinellosis in Argentina. Review of the casuistry registered from 1990 to 1999. Parasite. 2001;8(2 Suppl):S78–80. PubMed

Costantino SN, Gentile T, Venturiello SM. Immunoparasitological evaluation of *Trichinella spiralis* infection during human pregnancy: a small case series. Trans R Soc Trop Hyg. 2008;102:662–8. PubMed doi:10.1016/j.trstmh.2008.03.009

Rossi L, Coca F, Cricelli C, Troncoso A. First case of trichinosis caused by consumption of undercooked horse meat. J Infect Develop Countries. 2007;1:217–9.

Chile

García E, Mora L, Torres P, Jercic MI, Mercado R. First record of human trichinosis in Chile associated with consumption of wild boar (*Sus scrofa*). Mem Inst Oswaldo Cruz. 2005;100:17–8. PubMed doi:10.1590/S0074-02762005000100003

Morales MA, Vasquez J, Luengo J. Distribution and prevalence of trichinosis in man and pigs in Chile (1989–1995). J Parasitol Dia. 1999;23:63–5.

Schenone H, Olea A, Schenone H, Contreras MC, Mercado R, Sandoval L, et al. Current epidemiological situation of trichinosis in Chile. 1991–2000. Rev Med Chil. 2002;130:281–5. PubMed

Eastern Mediterranean Region

Iran

Kia EB, Meamar AR, Zahabiun F, Soodbakhsh A, Kordbacheh P. An outbreak of human trichinellosis due to the consumption of boar meat infected with *Trichinella* sp. [in Iranian]. Iran J Infect Dis Trop Med. 2008;41:35–8.

Lebanon

Haim M, Efrat M, Wilson M, Schantz PM, Cohen D, Shemer J. An outbreak of *Trichinella spiralis* infection in southern Lebanon. Epidemiol Infect. 1997;119:357–62. PubMed doi:10.1017/S0950268897007875

European Region

Belarus

Ivanov KS, Antonov VS, Knysh GG, Khadzhaeva AN, Antykova LP, Lavrova VP, et al. The clinical characteristics of 2 outbreaks of trichinelliasis [in Russian]. Med Parazitol (Mosk). 1990;4:41–2. PubMed

Skripova LV, Kovchur VN. Trichinellosis in Byelorussia. Wiad Parazytol. 1994;40:389–91. PubMed

Bosnia-Herzegovina

Cuperlovic K, Djordjevic M, Pavlovic S. Re-emergence of trichinellosis in southern Europe due to political and economic changes. Vet Parasitol. 2005;132:159–66. PubMed doi:10.1016/j.vetpar.2005.05.047

Bulgaria

Kurdova-Mintcheva R, Jordanova D, Ivanova M. Human trichinellosis in Bulgaria—epidemiological situation and trends. Vet Parasitol. 2009;159:316–9. PubMed

Petkova S, Mihov L, Vutova K, Tsenov I, La Rosa G, Pozio E. Epidemiological and clinical patterns of trichinellosis in Bulgaria from 1995 to 2002. Parasite. 2008;15:86–8. PubMed

Croatia

Aleraj B. Actual epidemiology of trichinellosis in Croatia [in Croatian]. Croatian Journal of Public Health 2008;4(14). http://www.hcjz.hr/sadrzaj.php?broj_casopisa=14

Croatian National Institute of Public Health. 2010. http://www.hzjz.hr/epocetna

Cvitović A, Miletic-Medved M, Gjenero-Margan I. An epidemic of trichinellosis in autumn 2004 in Slavonski Brod [in Croatian]. Acta Med Croatica. 2007;61:215–8. PubMed

Marinculić A, Gaspar A, Duraković E, Pozio E, La Rosa G. Epidemiology of swine trichinellosis in the Republic of Croatia. Parasite. 2001;8(2 Suppl):S92–4. PubMed

Puljiz I, Beus A, Kuzman I, Seiwerth S. Electrocardiographic changes and myocarditis in trichinellosis: a retrospective study of 154 patients. Ann Trop Med Parasitol. 2005;99:403–11. PubMed doi:10.1179/136485905X36307

Venus M, Puntaric D, Grgic M, Gmajnic R, Miskulin M. The effect of pest control on the incidence of trichinosis in Virovitica-Podravina County, Croatia. Vet Parasitol. 2008;156:226–33. PubMed doi:10.1016/j.vetpar.2008.06.010

Vojniković B, Brncić N, Zamolo G, Budiselić R, Njirić S, Novak S. Severe trichinellosis cured with pulse doses of glucocorticoids. Coll Antropol. 2001;25 Suppl:131–5. PubMed

Czech Republic

National Reference Laboratory for Tissue Helminthosis of the Czech Republic 1986–2009 [email: Libuse.kolarova@lfl.cuni.cz]

Estonia

Epshtein J. Estonian trichinellosis morbidity data 1985–2009. Dep. Communicable Disease Surveillance and Control. [email: jevgenia.epstein@terviseamet.ee]

France

Ancelle T, Dupouy-Camet J, Desenclos JC, Maillot E, Charlet F, Gravelat-Desclaux C, et al. Epidémie de trichinellose (France, 1993) bilan des investigations. Bulletin Épidémiologique Hebdomadaire. 1994;29:127–9.

Ancelle T, Dupouy-Camet J, Desenclos JC, Maillot E, Savage-Houze S, Drucker FCJ, et al. A multifocal outbreak of trichinellosis linked to horse meat imported from North America to France in 1993. Am J Trop Med Hyg. 1998;59:615–9. PubMed

Ancelle T, De Bruyne A, Niang M, Poisson DM, Prazuck T, Fur A, et al. Epidémie de trichinellose à *Trichinella nativa* due à la consommation de viande d'ours, France 2005. Bulletin Épidémiologique Hebdomadaire. 2006;14:96–8.

Basset D, Thiebaut MM, Pratlong F, Abraham B, Moryoussef A, Baldet P, et al. Epidémies familiales de trichinose dues a lingestion de sanglier sauvage dans la region Languedoc. Bulletin Épidémiologique Hebdomadaire. 1995;44:195.

Bernard E, Ozouf N, Toussauint-Gari M, Marty P, Pozio E, Le Fichoux Y, et al. Two new outbreaks of trichinosis [in French]. Med Mal Infect. 1995;25:611–4. doi:10.1016/S0399-077X(05)81038-8

De Bruyne A, Ancelle T, Vallée I, Boireau P, Dupouy-Camet J. Human trichinellosis from wild boar meat: a continuing parasitic risk in France. Euro Surveill. 2006;11: E060914.5. PubMed

Dupouy-Camet J, Allegretti S, Truong TP. Enquete sur l'incidence de la trichinellose en France (1994–1995). Bulletin Épidémiologique Hebdomadaire. 1998;28:122–3.

Dupouy-Camet J, Ancelle T, Talabani H. Surveillance de la trichinellose humaine en France. Rapport du CNR des Trichinella, 1 janvier 2009 - 31 decembre 2009. Rapport du CNR des Trichinella 2010.

Durant J, Toussaint-Gari M, Bernard E, Marty P, Le Fichoux Y, Dellamonica P. Epidémie familiare de trichinose. Sem Hop Paris. 1991;67:1507–12.

Gaillard T, Martinaud C, Kérébel S, Cellarier G, Muzellec Y, Brisou P. A propos de deux cas de trichinellose à *Trichinella britovi.* Ann Biol Clin (Paris). 2007;65:308–12. PubMed

Gari-Toussaint M, Bernard E, Quaranta JF, Marty P, Soler C, Ozouf N, et al. First report in France of an outbreak of human trichinellosis due to *Trichinella britovi*. In: Campbell WC, Pozio E, Bruschi F, editors. Trichinellosis. Proceedings of the 8th International Conference on Trichinellosis, 1994. Rome: Instituto Superiore di Sanita Press; 1994. p. 465–8.

Gari-Toussaint M, Tieulié N, Baldin JL, Marty P, Dupouy-Camet J, Delaunay P, et al. Trichinellose à *Trichinella britovi* dans les Alpes-Maritimes après consommation de viande de sanglier congelée, automne 2003. Bulletin Épidémiologique Hebdomadaire. 2004;21:87–8.

Gari-Toussaint M, Tieulié N, Baldin JL, Dupouy-Camet J, Delaunay P, Fuzibet JG, et al. Human trichinellosis due to *Trichinella britovi* in southern France after consumption of frozen wild boar meat. Euro Surveill. 2005;10:117–8. PubMed

Haeghebaert S, Servat M, Duchen C, Minet JC, Agrech AE, Thiese I, et al. Outbreak of trichinellosis in the Midi-Pyrénées region of France January–March 1998. Euro Surveill. 1998;3:83–5. PubMed

Hemery C, Haeghebaert S. New outbreak of trichinellosis in the Midi-Pyrénées region of France, September–October 1998. Euro Surveill. 1999;4:13–4. PubMed

Laurichesse H, Cambon M, Perre D, Ancelle T, Mora M, Hubert B, et al. Outbreak of trichinosis in France associated with eating horse meat. Commun Dis Rep CDR Rev. 1997;7:R69–73.

Maillot E, Desenclos JC, Dupouy-Camet J, Aubert P. Bulletin Épidémiologique Hebdomadaire. 1997;49:217–8.

Ranque S, Faugère B, Pozio E, La Rosa G, Tamburrini A, Pelissier JF, et al. *Trichinella pseudospiralis* outbreak in France. Emerg Infect Dis. 2000;6:543–7. PubMed doi:10.3201/eid0605.000517

Roumier M, Milhe P, Hautefort B, Benoist B. Four cases of trichinosis in Camargue (France) by consumption of meat of boar [in French]. Med Mal Infect. 1992;22:947–8. doi:10.1016/S0399-077X(05)80639-0

Soulé C. Trichinellose in France (1988–1990). Wiad Parazytol. 1992;38:153. PubMed

Soulé C. Trichinellose in France (1991–1992). Wiad Parazytol. 1994;40:394. PubMed

Georgia

Ivanov KS, Antonov VS, Knysh GG, Khadzhaeva AN, Antykova LP, Lavrova VP, et al. The clinical characteristics of 2 outbreaks of trichinelliasis [in Russian]. Med Parazitol (Mosk). 1990;Jul–Aug:41–2. PubMed

Germany

Anonymous. Human cases of trichinellosis in Germany (1965–2008). http://beck-online.beck.de/default.aspx?bcid=Y-100-G-BSeuchG

Jansen A, Schöneberg I, Stark K, Nöckler K. Epidemiology of trichinellosis in Germany, 1996–2006. Vector Borne Zoonotic Dis. 2008;8:189–96. PubMed doi:10.1089/vbz.2007.0183

Littmann M, Nöckler K, Hallauer J. Cluster of trichinellosis cases in Mecklenburg-Vorpommern, Germany. Euro Surveill. 2006;11: E060518.1.

Nöckler K, Wichmann-Schauer H, Hiller P, Müller A, Bogner K. Trichinellosis outbreak in Bavaria caused by cured sausage from Romania, January 2007. Euro Surveill. 2007;12: E070823.2.

Nothdurft HD, Brommer M, Eichenlaub D, Löscher T. A small outbreak of trichinosis in Germany caused by imported smoked ham [in German]. Dtsch Med Wochenschr. 1995;120:173–6. PubMed doi:10.1055/s-2008-1055330

Robert Koch Institute. InstitutSurStat@RKI, 2010. Human cases of trichinellosis in Germany from 2001 to 12 May 2010.

Greece

Boutsini S. Report on a trichinellosis outbreak in 2009. Pers. comm. [email: sboutsini@yahoo.gr].

Hungary

Glatz K, Danka J, Kucsera I, Pozio E. Human trichinellosis in Hungary from 1995 to 2009. Parasite. 2010;17:193–8. PubMed

Ireland

McHugh G, Kiely D, Low J, Healy ML, Hayes C, Clarke S. Importation of Polish trichinellosis cases to Ireland, June 2007. Euro Surveill. 2007;12: E070719.3. PubMed

Israel

Hefer E, Rishpon S, Volovik I. Trichinosis outbreak among Thai immigrant workers in the Hadera sub-district [in Hebrew]. Harefuah. 2004;143:656–60. PubMed

Marva E, Markovics A, Gdalevich M, Asor N, Sadik C, Leventhal A. Trichinellosis outbreak. Emerg Infect Dis. 2005;11:1979–81. PubMed

Italy

Di Bari C, Santagada G, Pozio E, Schiraldi O. Epidemiological research on trichinellosis in Apulia and Basilicata (southern Italy). Eur J Epidemiol. 1990;6:412–5. PubMed doi:10.1007/BF00151717

Frongillo RF, Baldelli B, Pozio E, Crapa G, Di Giuli C, Santirocchi M, et al. Report on an outbreak of trichinellosis in central Italy. Eur J Epidemiol. 1992;8:283–8. PubMed doi:10.1007/BF00144815

Pagni P, Bindi M, Burzagli L, Melchior M. An outbreak of trichinellosis in central Italy caused by pork meat ingestion. In: Campbell WC, Pozio E, Bruschi F, editors. Trichinellosis. Proceedings of the

8th International Conference on Trichinellosis, 1994. Rome: Instituto Superiore di Sanita Press; 1994. p. 487–90.

Pozio E, Santagada G, Di Bari C. Outbreak of trichinellosis in southern Italy. Trans R Soc Trop Med Hyg. 1986;80:997–8. PubMed doi:10.1016/0035-9203(86)90291-9

Pozio E, Cappelli O, Marchesi L, Valeri P, Rossi P. Third outbreak of trichinellosis caused by consumption of horse meat in Italy. Ann Parasitol Hum Comp. 1987;63:48–53.

Pozio E, Varese P, Goméz Morales MA, Croppo GP, Pelliccia D, Bruschi F. Comparison of human trichinellosis caused by *Trichinella spiralis* and *Trichinella britovi.* Am J Trop Med Hyg. 1993;48:568–75. PubMed

Pozio E, Sacchini D, Boni P, Tamburrini A, Alberici F, Paterlini F. Human outbreak of trichinellosis associated with the consumption of horsemeat in Italy. Euro Surveill. 1998;3:85–6.

Pozio E, La Rosa G, Gomez Morales MA. Epidemiology of human and animal trichinellosis in Italy since its discovery in 1887. Parasite. 2001;8:S106–8. PubMed

Pozio E, Marucci G. *Trichinella*-infected pork products: a dangerous gift. Trends Parasitol. 2003;19:338. PubMed doi:10.1016/S1471-4922(03)00138-7

Pozio E, Mesina P, Sechi F, Pira M, Liciardi M, Cossu P, et al. Human out break of trichinellosis in the Mediterranean island of Sardinia, Italy. Vet Parasitol. 2006;140:177–80. PubMed doi:10.1016/j.vetpar.2006.03.012

Pozio E, Cossu P, Marucci G, Amati M, Ludovisi A, Gomez Morales MA, et al. The birth of a *Trichinella britovi* focus on the Mediteranean island of Sardinia (Italy). Vet Parasitol. 2009;159:361–3. PubMed

Kyrgyzstan

Witschi M. WHO Country Office of Kyrgyzstan 2010 [email: wim@euro.who.int].

Latvia

Public Health Agency Annual Report of Latvia [email: gunita.deksne@bior.gov.lv].

Lithuania

Bartuliene A, Jasulaitiene V, Malakauskas A. Human trichinellosis in Lithuania, 1990–2004. Euro Surveill. 2005;10: E050714.6. PubMed

Bartuliene A, Liausediene R, Motiejuniene V. Trichinellosis outbreak in Lithuania, Ukmerge region, June 2009. Euro Surveill. 2009;14:24. PubMed

EpiNorth. http://www.epinorth.org/eway/default.aspx?pid=230&trg=Area_5279&MainArea_5260=5279:0:15,2937:1:0:0:0:0&Area_5279=5291:44530:1:5290:1:0:0&diseaseid=38

Malakauskas A. molecular epidemiology of trichinellosis in Lithuania and biological characteristics of *Trichinella* genotypes in rat. PhD Thesis, Copenhagen, Denmark: The Royal Veterinary and Agricultural University; 2002. p. 1–135.

Rockiene A. Trichinellosis in Lihuania. 2nd Nordic-Baltic congress on Infectious Diseases “Prevention in Focus.” Latvia 1996, p. 38–39.

Macedonia

Hristovski M, Dzimrevski J, Bosnakovski J, Markovski G, Ancevski V, Krstic I. Trichinellosis in the Republic of Macedonia. Makedonski Veterinaren Pregled. 1992;21:85–90.

Poland

Adonajlo A. Trichinellosis in Poland in 1986 [in Polish]. Przegl Epidemiol. 1988;42:113–5. PubMed

Adonajlo A. Trichinellosis in Poland in 1987 [in Polish]. Przegl Epidemiol. 1989;43:107–9. PubMed

Adonajlo A. Trichinellosis in Poland in 1988 [in Polish]. Przegl Epidemiol. 1990;44:119–23. PubMed

Adonajlo A. Trichinellosis in Poland in 1989 [in Polish]. Przegl Epidemiol. 1991;45:113–5. PubMed

Adonajlo A. Trichinellosis in Poland in 1990 [in Polish]. Przegl Epidemiol. 1992;46:127–30. PubMed

Adonajlo A. Trichinellosis in Poland in 1991 [in Polish]. Przegl Epidemiol. 1993;47:161–4. PubMed

Koncki A. Trichinellosis in 1992 [in Polish]. Przegl Epidemiol. 1994;48:145–7. PubMed

Przybylska A. Trichinellosis in Poland in 2000 [in Polish]. Przegl Epidemiol. 2002;56:353–6. PubMed

Przybylska A. Trichinellosis in Poland in 2001 [in Polish]. Przegl Epidemiol. 2003;57:153–8. PubMed

Przybylska A. Trichinellosis in Poland in 2002 [in Polish]. Przegl Epidemiol. 2004;58:157–64. PubMed

Sadkowska-Todys M, Gołab E. Trichinellosis in Poland in 2003 [in Polish]. Przegl Epidemiol. 2005;59:327–30. PubMed

Sadkowska-Todys M, Gołab E. Trichinellosis in Poland in 2004 [in Polish]. Przegl Epidemiol. 2006;60:503–7. PubMed

Sadkowska-Todys M, Gołab E. Trichinellosis in Poland in 2005 [in Polish]. Przegl Epidemiol. 2007;61:301–4. PubMed

Sadkowska-Todys M, Gołab E. Trichinellosis in Poland in 2008 [in Polish]. Przegl Epidemiol. 2008;62:347–50. PubMed

Sadkowska-Todys M, Gołab E. Trichinellosis in Poland in 2007 [in Polish]. Przegl Epidemiol. 2009;63:263–6. PubMed

Seroka D. Trichinellosis in Poland in 1993 [in Polish]. Przegl Epidemiol. 1995;49:181–7. PubMed

Seroka D. Trichinellosis in Poland in 1994 [in Polish]. Przegl Epidemiol. 1996;50:191–7. PubMed

Seroka D. Trichinellosis in Poland in 1995 [in Polish]. Przegl Epidemiol. 1997;51:157–60. PubMed

Seroka D. Trichinellosis in Poland in 1996 [in Polish]. Przegl Epidemiol. 1998;52:133–7. PubMed

Seroka D. Trichinellosis in Poland in 1997 [in Polish]. Przegl Epidemiol. 1999;53:155–8. PubMed

Seroka D. Trichinellosis in Poland in 1998 [in Polish]. Przegl Epidemiol. 2000;54:175–9. PubMed

Seroka D. Trichinellosis in Poland in 1999 [in Polish]. Przegl Epidemiol. 2001;55:155–8. PubMed

Romania

Blaga R, Durand B, Antoniu S, Gherman C, Cretu C, Cozma V, et al. A dramatic increase in the incidence of human trichinellosis in Romania over the past 25 years: impact of political changes and regional food habits. Am J Trop Med Hyg. 2007;76:983–6. PubMed

Neghina R, Neghina AM, Marincu I, Moldovan R, Iacobiciu I. Epidemiological and diagnostic findings during a 16-year-long trichinellosis surveillance in Timis County, Romania. Vet Parasitol. 2009a;159:328–31. PubMed

Neghina R, Neghina AM, Marincu I, Moldovan R, Iacobiciu I. Trichinellosis, a threatening and re-emerging disease in a Romanian western county. Vector Borne Zoonotic Dis. 2009b;9:717–21. PubMed doi:10.1089/vbz.2008.0204

Neghina R, Neghina AM, Marincu I, Moldovan R, Iacobiciu I. Epidemiology and epizoology of trichinellosis in Romania 1868–2007. Vector Borne Zoonotic Dis. 2010a;10:323–8. PubMed doi:10.1089/vbz.2009.0084

Neghina R, Neghina AM, Marincu I, Moldovan R, Iacobiciu I. Evidence of *Trichinella spiralis* in Timis county, Romania: a report of a winter trichinellosis outbreak in 2008 due to consumption of contaminated pork. Vector Borne Zoonotic Dis. 2010b;10:931–3. PubMed doi:10.1089/vbz.2009.0057

Nemet C, Rogozea L, Dejica R. Results of the follow-up of the former trichinosis patients from Brasov county—Romania. Vet Parasitol. 2009;159:320–3. PubMed

Russia

Ozeretskovskaya NN, Mikhailova LG, Sabgaida TP, Dovgalev AS. New trends and clinical patterns of human trichinellosis in Russia at the beginning of the XXI century. Vet Parasitol. 2005;132:167–71. PubMed doi:10.1016/j.vetpar.2005.05.056

Serbia

Cuperlovic K, Djorjevic M, Pavlovic S, Sofronic-Milosavljevic L. Present status of trichinellosis in Yugoslavia: Serbia. Parasite. 2001;8(2 Suppl):S95–7. PubMed

Cuperlovic K, Djordjevic M, Pavlovic S. Re-emergence of trichinellosis in southern Europe due to political and economic changes. Vet Parasitol. 2005;132:159–66. PubMed doi:10.1016/j.vetpar.2005.05.047

Djordjevic M, Bacic M, Petricevic M, Cuperlovic K, Malakauskas A, Kapel CMO, et al. Social, political, and economic factors responsible for the re-emergence of trichinellosis in Serbia: a case study. J Parasitol. 2003;89:226–31. PubMed doi:10.1645/0022-3395(2003)089[0226:SPAEFR]2.0.CO;2

Slovakia

Dubinský P, Stefancikova A, Hovorka I, Mitterpark J. Epidemiology of trichinellosis in Slovakia. In: Campbell WC, Pozio E, Bruschi F, editors. Trichinellosis. Proceedings of the 8th International Conference on Trichinellosis, 7–10 September, 1993. Orvieto, Italy, p. 515–520.

Dubinský P, Stefanciková A, Kinceková J, Ondriska F, Reiterová K, Medvedová M. Trichinellosis in the Slovak Republic. Parasite. 2001;8(2 Suppl):S100–2. PubMed

Paraličová Z, Kinčeková J, Schréter I, Jarčuska P, Dubinský P, Porubčin S, et al. Outbreak of trichinellosis in eastern Slovakia. Helminthologia. 2009;46:209–13. doi:10.2478/s11687-009-0039-2

Reiterová K, Kinceková J, Snábel V, Marucci G, Pozio E, Dubinský P. *Trichinella spiralis*–outbreak in the Slovak Republic. Infection. 2007;35:89–93. PubMed doi:10.1007/s15010-007-6122-z

Slovenia

Brglez J. A new epidemic focus on trichinellosis [in Slovenian]. Veterinarske Novice. 1996;22:135–6.

Saletinger R, Rejc-Marko J, Unuk S, Logar J. Trichinosis—case presentation [in Slovenian]. Zdrav Vestn. 2007;76:175–8.

Spain

Anonymous. Brotes de triquinosis. Espana. Temporada 1985/1986. Boletin Epidemiologico Semanal. 1986; N. 1748.

Anonymous. Brotes de triquinosis. Espana. Temporada 1986–1987. Boletin Epidemiologico Semanal. 1987; N. 1779.

Anonymous. Brotes de triquinosis. Espana. Temporadas 1987–1988. Boletin Epidemiologico Semanal. 1988; N. 1823.

Anonymous. Brotes de triquinosis. Espana. Temporadas 88/89 Y 89/90. Boletin Epidemiologico Semanal. 1990; N. 1857.

Anonymous. Vigilancia de la triquinosis en Espana. Boletin Epidemiologico Semanal. 1995;3:105–12.

Anonymous. Number of trichinellosis cases in Spain from 1986 to 2009. tgarate@isciii.es; 2010.

Arévalo A, Bringas MJ, Rodriguez R, Menor A. Description of a trichinosis outbreak in the province of Salamanca [in Spanish]. Rev Esp Quimioter. 2009;22:115–6. PubMed

Benitez JA, Cruz JM, Adrados G, Gonzalez T. Triquinosis: a proposito de 17 casos aparecidos en un mismo brote. Enferm Infecc Microbiol Clin. 1987;5:122–3.

Cobo J, Gómez Cerezo J, Medraño JC, Zapico R, Cruz Martinez A, Molina F, et al. Trichinosis. A study of a large outbreak on the Iberian peninsula [in Spanish]. An Med Interna. 1991;8:441–4.

Cortés Blanco M, Garcia-Cabanas A, Guerra-Peguero F, Ramos-Aceitero JM, Herrera-Guibert D, Martinez-Navarro JF. Outbreak of trichinellosis in Caceres, Spain, December 2001–February 2002. Euro Surveill. 2002;7:136–8. PubMed

de la Cruz de Julián I, Díaz García JM, Alvarez Lana P, García Colmenero C. An outbreak of trichinosis in Huerta del Marquesado (the Cañete-Cuenca basic health area). December 1992 to January 1993 [in Spanish]. Rev Sanid Hig Publica (Madr). 1994;68:513–20.

de la Torre Cecilia C, Espino Aguilar R, Cárdenas Talaverón C, Cañuelo Ruiz O, Garrido Palomo R, Baena Sáez J, et al. Trichinosis: presentation of 2 cases [in Spanish]. An Esp Pediatr. 1989;30:227–8. PubMed

Gallardo MT, Mateos L, Artieda J, Wesslen L, Ruiz C, Garcia MA, et al. Outbreak of trichinellosis in Spain and Sweden due to the consumption of wild boar meat contaminated with *Trichinella britovi*. Euro Surveill. 2007;12:E070315.1. PubMed

Gomez-Garcia V, Hernandez-Quero J, Rodriguez-Osorio M. Short report: human infection with *Trichinella britovi* in Granada, Spain. Am J Trop Med Hyg. 2003;68:463–4. PubMed

Herráez García J, Leon Garcia LA, Lanusse Senderos C, Cortés Blanco M, García Cabanas A. Outbreak of trichinellosis in the region of la Vera (Carceres, Spain) caused by *Trichinella britovi* [in Spanish]. An Med Interna. 2003;20:63–6. PubMed doi:10.4321/S0212-71992003000200003

López Hernández B, Velázquez de Castro MT, Galicia García MD, Sabonet JC. Outbreak of *Trichinella britovi* infection in Granada in the spring of 2000 [in Spanish]. Rev Esp Salud Publica. 2001;75:467–73. PubMed doi:10.1590/S1135-57272001000500007

Mangas Gallardo I, Tello Anchuela O. Vigilancia de la triquinosis en Espana. Temporadas 1989/90–1992/93. Bol Epidemiol Microbiol. 1994;1:233–4.

Martinez Corral JJ, Esteban Niveiro MJ, Oliet Pala R. Triquinosis producida por el consumo de carne de porcino infestado con *Trichinella spiralis*. Investigation y estudio de un brote alimentario. Salud Publica. 2000;12:36–40.

Rodriguez-Osorio M, Abad JM, De Haro T, Villa-Real R, Gomez Garcia V. Human trichinellosis in southern Spain: serologic and epidemiologic study. Am J Trop Med Hyg. 1999;61:834–7. PubMed

Serrano R, Lacasa J, Velazquez J, Ziad F, Aznar R. Triquinosis: nuevo brote epidémico por ingesta de embutido de jabali. Enferm Infecc Microbiol Clin. 1989;7:428–31. PubMed

Tiberio G, Lanzas G, Galarza MI, Sanchez J, Quilez I, Martinez Artola V. Short report: an outbreak of trichinosis in Navarra, Spain. Am J Trop Med Hyg. 1995;53:241–2. PubMed

Tiberio G, Rivero M, Lanzas G, Redin D, Ardanaz E, Fernandez C, et al. Trichinellosis: study of 2 outbreaks in Navarre [in Spanish]. Enferm Infecc Microbiol Clin. 1997;15:151–3. PubMed

Switzerland

Lozano-Becerra J.C., pers. comm. [email: JuanCarlos.Lozano-Becerra@eoc.ch].

Lupinc L, Markwalder K, Nigg C. Eosinophilie bei einer familie aus Bosnien. Praxis. 2003;92:2212–6. PubMed doi:10.1024/0369-8394.92.51.2212

Turkey

Akkoc N, Kuruuzum Z, Aker S, Yuce A, Onen F, Yapar N, et al. A large-scale out break of trichinellosis caused by *Trichinella britovi* in Turkey. Zoonoses Public Health. 2009;56:65–70. PubMed doi:10.1111/j.1863-2378.2008.01158.x

Heper Y, Yilmaztepe F, Komitova R, Akalin H, Vutova K, Helvaci S. A trichinosis outbreak caused by wild boar meat in Turkey. Parasite. 2005;12:191–2. PubMed

United Kingdoom

Milne LM, Bhagani S, Bannister BA, Laitner SM, Moore P, Eza D, et al. Trichinellosis acquired in the United Kingdom. Epidemiol Infect. 2001;127:359–63. PubMed doi:10.1017/S0950268801005994

Ukraine

Ministry of Healthcare of Ukraine. Trichinellosis in Ukraine from 1986 to 2010. Central health-epidemiology station of Ministry of healthcare of Ukraine. 2010.

South-East Asia Region

Thailand

Charkrit S. Study on clinical manifestations of trichinosis in Payao province. Communicable Diseases Journal. 1998;24:242–7.

Chotmongkol V, Intapan PM, Koonmee S, Kularbkaew C, Aungaree T. Case report: acquired progressive muscular hypertrophy and trichinosis. Am J Trop Med Hyg. 2005;72:649–50. PubMed

Limsuwan S, Siriprasert V. A clinical study on trichinosis in Changwat Phayao, Thailand. Southeast Asian J Trop Med Public Health. 1994;25:305–8. PubMed

Jongwutiwes S, Chantachum N, Kraivichian P, Siriyasatien P, Putaporntip C, Tamburrini A, et al. First outbreak of human trichinellosis caused by *Trichinella pseudospiralis*. Clin Infect Dis. 1998;26:111–5. PubMed doi:10.1086/516278

Khumjui C, Choomkasien P, Dekumyoy P, Kusolsuk T, Kongkaew W, Chalamaat M, et al. Outbreak of trichinellosis caused by *Trichinella papuae*, Thailand. 2006. Emerg Infect Dis. 2008;14:1913–5. PubMed

Watt G, Saisorn S, Jongsakul K, Sakolvaree Y, Chaicumpa W. Blinded, placebo-controlled trial of antiparasitic drugs for trichinosis myositis. J Infect Dis. 2000;182:371–4. PubMed doi:10.1086/315645

India

Alipuria S, Sangha HK, Singh G, Pandhi S. Trichinosis—a case report. Indian J Pathol Microbiol. 1996;39:231–2. PubMed

Handa R, Aggarwal P, Sarkar C, Vijayarghavan M, Mattewal A, Arya V, et al. A patient with muscle weakness. J Indian Rheum Assoc. 2000;8:85–7.

Mohan H, Aggarwal R, Nada R, Punia RPS, Ahluwalia M. Trichinosis of psoas muscle. J Assoc Physicians India. 2002;50:729–30. PubMed

Western Pacific Region

China

Ci R, Si T, Wang HJ. An outbreak of trichinellosis in Milin County of Tibet Autonomous Region [in Chinese]. Zhongguo Ji Sheng Chong Xue Yu Ji Sheng Chong Bing Za Zhi. 2003;21:60.

Cui J, Wang ZQ, Wu F, Jin XX. Epidemiological and clinical studies on an outbreak of trichinosis in central China. Ann Trop Med Parasitol. 1997;91:481–8. PubMed doi:10.1080/00034989760842

Wang ZQ, Cui J. The epidemiology of human trichinellosis in China during 1964–1999. Parasite. 2001;8:S63. PubMed

Gong XH, Guo WM, Cirendunzhu, Long EK, Ma Y, Bianbazuoma, et al. Investigation on an outbreak of trichinosis and clinical analysis [in Chinese]. Zhongguo Ji Sheng Chong Xue Yu Ji Sheng Chong Bing Za Zhi. 2008;26(1):79–80.

Lo YC, Hung CC, Lai CS, Wu Z, Nagano I, Maeda T, et al. Human trichinosis after consumption of soft-shelled turtles, Taiwan. Emerg Infect Dis. 2009;15:2056–8. PubMed

Xu B, Chang J, Gao G, Tang B, Hong J, Chen Y, et al. Four outbreaks of human trichinellosis in Henan Province. Chin Med J (Engl). 1995;108:872–4. PubMed

Ye P, Jianhua H, Zhengkun Z, Desong G, Hao W. The investigation over an outbreak of trichinellosis caused by eating *Sus scrofa.* Chinese Journal of Zoonoses. 2003;19:12.

Japan

Kusuhara Y, Maeno Y, Nagase K, Taniguchi K, Torikai K, Takahashi Y. A case of mixed infection with *Schistosoma haematobium* and *Trichinella sp.* Kansenshogaku Zasshi. 1999;73(6):614–7.

Nakamura T, Miura T, Nakaoka T, Nagano I, Takahashi Y, Iwamoto A. A case of trichinellosis with spontaneous remission [in Japanese]. Kansenshogaku Zasshi. 2003;77:839–43.

Shiota T, Arizono N, Yoshioka T, Ishikawa Y, Fujitake J, Fujii H, et al. Imported trichinellosis with severe myositis—report of a case [in Japanese]. Kansenshogaku Zasshi. 1999;73(1):76–82.

Yoshikawa M, Ouji Y, Moriya K, Saito K, Ishizaka S, Sukurai M, et al. Immunopositivity for *Trichinella spiralis* presenting in a case of paragonimiasis wetermani-suspected infection by ingestion of raw bear meat. J Nara Med Assoc. 2005;56:253–9.

Korea

Lee HC, Kim HJ, Oh HY, Kim JH, Kim HG, Lee MS, et al. A case of trichinosis caused by eating a wild badger. Korean Journal of Medicine 1999;56:134–8.

Sohn WM, Huh S, Chung DI, Pozio E. Molecular identification of Korean *Trichinella* isolates. Korean J Parasitol. 2003;41:125–7. PubMed doi:10.3347/kjp.2003.41.2.125

Kim E, Pyun RH, Park JH, Kim KH, Choi I, Park HH, et al. Family outbreak of trichinosis after eating a raw meat of wild swine. Korean J Parasitol. 2003;35:180–4.

Laos

Barennes H, Sayasone S, Odermatt P, Bruyne AD, Sitthivone HS, Newton PN, et al. A major trichinellosis outbreak suggesting a high endemicity of *Trichinella* infection in northern Laos. Am J Trop Med Hyg. 2008;78:40–4. PubMed

Sayasone S, Odermatt P, Vongphrachanh P, Keoluangkhot N, Dupouy-Camet J, Newton PN, et al. Trichinellosis outbreak in Borikhamxay Province, Lao PDR. Trans R Soc Trop Med Hyg. 2006;100:1126–9. PubMed doi:10.1016/j.trstmh.2006.01.010

Suwansrinon K, Wilde H, Burford B, Hanvesakul R, Visith Sitprija V. Human trichinellosis from Laos. J Travel Med. 2007;14:274–7. PubMed doi:10.1111/j.1708-8305.2007.00136.x

Taybouavone T, Odermatt P, Keoluangkhot V, Delanos-Gregoire N, Dupouy-Camet J, Strobel M, et al. Trichinellosis during pregnancy: a case control study in the Lao People's Democratic Republic. Vet Parasitol. 2009;159:332–6. PubMed

Singapore

Kurup A, Yew WS, San LM, Ang B, Lim S, Tai GK. Outbreak of suspected trichinosis among travelers returning from a neighboring island. J Travel Med. 2000;7:189–93. PubMed doi:10.2310/7060.2000.00058

Vietnam

De NV, Toan ND, Son DT, Van P. Report an epidemic area of trichinellosis in Tuan Giao district, Lai Chau province. Proc 5th Seminar on Food- and Water-Borne Parasitic Zoonoses. Bangkok, Thailand. 2006Nov 28–30. p. 48.

Taylor WRJ, Tran GV, Nguyen TQ, Dang DV, Nguyen VK, Nguyen CT, et al. Acute febrile myalgia in Vietnam due to trichinellosis following the consumption of raw pork. Clin Infect Dis. 2009;49:e79–83. PubMed doi:10.1086/605533

Section B. Trichinellosis infections acquired in countries different from those where the disease was developed and diagnosed, 1986–2009

Country where the infection was developed and diagnosed	Country where infection was acquired	Period	No. clinical cases	References
Austria	Yugoslavia	2000–2006	10	Austrian Ministry of Health Report, 1986–2009
Czech Republic	Poland	1992–2006	2	National Reference Laboratory for Tissue Helminthosis
	Ukraine		2	
	France		1	
Denmark	Poland	2007	12	Stensvold et al., 2007
France	Algeria	1986	5	Michel et al., 1986
	Turkey	1994–1995	3	Dupouy-Camet et al., 1998
	Kenya		2	
	Greenland	1995	2	Nozais et al., 1996
	Laos	1991	1	Ancelle et al., 2006
	Yugoslavia	1996	1	
	Croatia	1999	1	
	Cameroon	1999	1	
	Spain	2001	1	
	Canada	2004	1	
	Thailand	2004	1	
	Laos	2005	3	
	Canada	2005	8	
	Algeria	2004	1	Nezri et al., 2006
	Laos	2007	1	Dupouy-Camet et al., 2008
	Senegal	2009	5	Dupouy-Camet et al., 2009
Belgium	Canada	2009	1	Houzé et al., 2009
People's Republic of China (Hong Kong Special Administrative Region)			1	
France			4	
Germany			1	
Germany	Poland	2007	3	Schmiedel and Kramme, 2007
Italy	Romania	2008	4	Angheben et al., 2008
Netherlands	Yugoslavia	1992	3	T. Kortbeek, pers. comm.
	Montenegro	1999	5	Pinelli et al., 2004
Total			86	

References

Ancelle T, De Bruyne A, Niang M, Poisson DM, Prazuck T, Fur A, et al. Epidémie de trichinellose à *Trichinella nativa* due à la consommation de viande d'ours, France 2005. Bulletin Épidémiologique Hebdomadaire. 2006;14:96–8.

Angheben A, Mascarello M, Zavarise G, Gobbi F, Monteiro G, Marocco S, et al. Outbreak of imported trichinellosis in Verona, Italy, January 2008. Euro Surveill. 2008;13:pii18891. PubMed

Austrian Ministry of Health Report 1986–2009, received by email from Dr. Herbert Auer, Dep. Med. Parasitol., Institute of Specific Prophylaxis and Tropical Medicine Center of Pathophysiology, Infectiology and Immunology Kinderspitalgasse 15 1090 Wien.

Dupouy-Camet J, Allegretti S, Truong TP. Enquete sur l'incidence de la trichinellose en France (1994–1995). Bulletin Épidémiologique Hebdomadaire. 1998;28:122–3.

Dupouy-Camet et al. Surveillance dela trichinellose humaine en France. Rapport du CNR des *Trichinella*, 1er janvier 2007–31 decembre 2007. 2008.

Dupouy-Camet J et al. Surveillance dela trichinellose humaine en France. Rapport du CNR des *Trichinella*, 1er janvier 2008–31 decembre 2008. 2009.

Houzé S, Ancelle T, Matra R, Boceno C, Carlier Y, Gajadhar AA, et al. Trichinellosis acquired in Nunavut, Canada in September 2009: meat from grizzly bear suspected. Euro Surveill. 2009;14:pii: 19383. PubMed

Koortbek T. [email: Titia.Kortbeek@rivm.nl].

Michel PH, Zurlinden A, Charvillat L, Levencq P, Janin G, Bayle A, et al. Five new cases of trichinosis [in French]. Presse Med. 1986;15:2073–4. PubMed

National Reference Laboratory for Tissue Helminthosis of the Czech Republic 1986–2009 [email: Libuse.kolarova@lfl.cuni.cz]

Nezri M, Ruer J, De Bruyne A, Cohen-Valensi R, Pozio E, Dupouy-Camat J. Premiere observation d'un cas de humain de trichinellose a *Trichinella britovi* en Algerie après consummation de viande de chacal (*Canis aureus*). Bull Soc Pathol Exot. 2006;99:94–5. PubMed

Nozais JP, Mannevy V, Danis M. Two cases of trichinosis from polar bear (*Thalarctos maritimus*) meat [in French]. Med Mal Infect. 1996;26:732–3. doi:10.1016/S0399-077X(96)80107-7

Pinelli E, Mommers M, Homan W, van Maanen T, Kortbeek LM. Imported human trichinellosis: sequential IgG4 antibody response to *Trichinella spiralis.* Eur J Clin Microbiol Infect Dis. 2004;23:57–60. PubMed doi:10.1007/s10096-003-1039-7

Schmiedel S, Kramme S. Cluster of trichinellosis cases in Germany, imported from Poland, June 2007. Euro Surveill. 2007;12:E070719.4. PubMed

Stensvold CR, Nielsen HV, Mølbak K. A case of trichinellosis in Denmark, imported from Poland, June 2007. Euro Surveill. 2007;12:E070809.3. PubMed

Section C. References for data on frequency of major clinical signs and symptoms in persons infected during 1986–2009

African Region

Ethiopia

Kefenie H, Bero G. Trichinosis from wild boar meat in Gojjam, north-west Ethiopia. Trop Geogr Med. 1992;44:278–80. PubMed

Kefenie H, Wolde H, Abuherpo M. Trichinosis from wild boar meat in Arsi, central Ethiopia. Ethiop Med J. 1988;26:97–100. PubMed

Region of the Americas

Canada

Ancelle T, Bruyne A, Nuang M, Poisson DM, Prazuck T, Fur A, et al. Epidemic of *Trichinella nativa* trichinosis caused by consumption of bear meat, France, 2005. Bull Epidemiol Hebd. 2006;14:96–8.

Greenbloom SL, Martin-Smith P, Issacs S, Marshall B, Kittle DC, Kain KC, et al. Outbreak of trichinosis in Ontario secondary to the ingestion of wild boar meat. Can J Public Health. 1997;88:52–6. PubMed

Heinzig L. Case reviews—botulism and trichinosis. EpiNorth. 1996;8:7.

Houzé S, Ancelle T, Matra R, Boceno C, Carlier Y, Gajadhar AA, et al. Trichinellosis acquired in Nunavut, Canada in September 2009: meat from grizzly bear suspected. Euro Surveill. 2009;14: pii: 19383. PubMed

MacLean JD, Poirier L, Gyorkos TW, Proulx JF, Bourgeault L, Corriveau A, et al. Epidemiologic and serologic definition of primary and secondary trichinosis in the Arctic. J Infect Dis. 1992;165:908–12. PubMed doi:10.1093/infdis/165.5.908

Møller LN, Petersen E, Kapel CMO, Melbye M, Koch A. Outbreak of trichinellosis associated with consumption of game meat in West Greenland. Vet Parasitol. 2005;132:131–6. PubMed doi:10.1016/j.vetpar.2005.05.041

Nozias JP, Mannevey V, Danis M. Two cases of trichinosis from polar bear (*Thalarctos maritimus*) meat. Med Mal Infect. 1996;26:732–3.

Schellenberg RS, Tan B, Irvine JD, Stockdale DR, Gajadhar AA, Serhir B, et al. An outbreak of trichinellosis due to consumption of bear meat infected with *Trichinella nativa*, in 2 northern Saskatchewan communities. J Infect Dis. 2003;188:835–43. PubMed doi:10.1086/378094

Serhir B, MacLean JD, Healey S, Forbes L. Outbreak of trichinellosis associated with arctic walruses in northern Canada, 1999. Can Commun Dis Rep. 2001; 27–04:31–6.

Viallet J, MacLean JD, Goresky CA, Staudt M, Routhier G, Law C. Arctic trichinosis presenting as prolonged diarrhea. Gastroenterology. 1986;91:938–46. PubMed

United States

Centers for Disease Control and Prevention. Trichinosis—Hawaii. MMWR Morb Mortal Wkly Rep. 1987;36:14–6. PubMed

Centers for Disease Control and Prevention. Trichinellosis associated with bear meat—New York and Tennessee, 2003. MMWR Morb Mortal Wkly Rep. 2004;53:606–10. PubMed

Dworkin MS, Gamble HR, Zarlenga DS, Tennican PO. Outbreak of trichinellosis associated with eating cougar jerky. J Infect Dis. 1996;174:663–6. PubMed doi:10.1093/infdis/174.3.663

Graves T, Harkness J, Crutcher JM. Case report: locally aquired trichinosis in an immigrant from Southeast Asia. J Okla State Med Assoc. 1996;89:402–4. PubMed

Kennedy ED, Hall RL, Montgomery SP, Pyburn DG, Jones JJ. Trichinellosis surveillance—United States, 2002–2007. MMWR Surveill Summ. 2009;58:1–7. PubMed

Landry SM, Kiser D, Overby T, Mays B, Caplen CW. Trichinosis: common source outbreak related to commercial pork. South Med J. 1992;85:428–9. PubMed doi:10.1097/00007611-199204000-00022

McAuley JB, Michelson MK, Hightower AW, Engeran S, Wintermeyer LA, Schantz PM. A trichinosis outbreak among Southeast Asian refugees. Am J Epidemiol. 1992;135:1404–10. PubMed

McAuley JB, Michelson MK, Schantz PM. Trichinellosis surveillance, United States, 1987–1990. MMWR CDC Surveill Summ. 1991;40:35–42. PubMed

Nelson M, Wright TL, Pierce A, Krogwold RA. A common-source outbreak of trichinosis from consumption of bear meat. J Environ Health. 2003;65:16–9, 24. PubMed

Petri WA, Holsinger JR, Pearson RD. Common-source outbreak of trichinosis associated with eating raw home-butchered pork. South Med J. 1988;81:1056–8. PubMed doi:10.1097/00007611-198808000-00029

Roy SL, Lopez AS, Schantz PM. Trichinellosis surveillance—United States, 1997–2001. MMWR Surveill Summ. 2003;52:1–8. PubMed

Mexico

Alverez-Chacón R, Riega-Carnero RE, Garcia-Rosales JJ, Wong-Chio M, Cob-Sosa CE. Triqinosis en el niño. Informe de 8 casos. Bol Med Hosp Infant Mex. 1992;49:286–90. PubMed

Hernández M, Ramos-Martinez E, Casco-Sanchez EJ, Morales-Gomez JM, Perez-Matos EM. Triquinos aguda. Epidemia de 166 casos en Cuidad Delicias, Chih. Diagnostico por compression tisular y tinicion. Gac Med Mex. 1992;128:45–50. PubMed

Santos Durán-Ortiz J, García-de la Torre I, Orozco-Barocio G, Martínez-Bonilla G, Rodríguez-Toledo A, Herrera-Zárate L. Trichinosis with severe myopathic involvement mimicking polymyositis. Report of a family outbreak. J Rheumatol. 1992;19:310–2. PubMed

Sartí Gutiérrez EJ, Gutierrez Ospina I, Kopman JS. Brote de triquinos ocurrido en una oficina de gobierno. Mexico, D.F. Salud Publica Mex. 1986;28:41–7. PubMed

Zamora-Chávez A. de la O-Cavasos ME, Bernal-Redondo RM, Berrones-Espericueta D, Vazquez-Antona C. Acute trichinosis in children. Intrafamilial epidemic outbreak in Mexico City [in Spanish]. Bol Med Hosp Infant Mex. 1990;47:395–400. PubMed

Argentina

Ambrosioni J, Cecchini D, Castellaro P, Biscione F, Lloveras S, Orduna T. Trichinellosis: epidemiological, clinical and laboratory aspects. A retrospective study (1994–2003). Enferm Infecc Microbiol Clin. 2006;24:440–4. PubMed doi:10.1157/13091782

Calcagno MA, Teixeira C, Forastiero MA, Coastantino SN, Venturiello SM. Aspectos clinicos, serologicos y parasitologicos de un brote de triquinos humana en Villa Mercedes, San Luis, Argentina. Medicina (B Aires). 2005;65:302–6. PubMed

Caminoa RA, Carbonell JA, Tapia N, Ledesma M. Brote de triquinosis humana en Necochea, Buenos Aires, Argentina. ACTA Bioquim Clin Latinamericana. 1995;29:493–8.

Caminoa RA, Ledesma M, Gallicchio O, Sanchez G, Benitez M. Human trichinosis in Buenos Aires province, Argentina during 1998. Vet-Investigacion Vet. 2000; 2:101–4.

Nuñez GG, Susana N, Costantino SN, Gentile T, Venturiello SM. Immunoparasitological evaluation of *Trichinella spiralis* infection during human pregnancy: a small case series. Trans R Soc Trop Med Hyg. 2008;102:662–8. PubMed doi:10.1016/j.trstmh.2008.03.009

Siciliano C, Nieto Sosa L, Chalub E, Shictong G. Nuevo brote de triquinosis en Cordoba (Rep. Argentina). Actual Infectologia (Montevideo). 1988;4:14–20.

Chile

García E, Mora L, Torres P, Jercic MI, Mercado R. First record of human trichinosis in Chile associated with consumption of wild boar (*Sus scrofa*). Mem Inst Oswaldo Cruz. 2005;100:17–8. PubMed doi:10.1590/S0074-02762005000100003

Zamorano CG, Contreras MC, Espinoza A, Paredes M, Sandoval L, Schulz E, et al. Brote de triquinosis en la Comuna de Purranque, X Region, Chile, Octubre–Noviembre, 1992. Bol Chil Parasitol. 1994;49:38–42. PubMed

Eastern Mediterranean Region

Iran

Kia EB, Meamar AR, Zahabiun F, Soodbakhsh A, Kordbacheh P. An outbreak of human trichinellosis due to the consumption of boar meat infected with *Trichinella* sp. [in Iranian]. Iran J Infect Dis Trop Med. 2008;41:35–48.

Lebanon

Haim M, Efrat M, Wilson M, Schantz PM, Cohen D, Shemer J. An outbreak of *Trichinella spiralis* infection in southern Lebanon. Epidemiol Infect. 1997;119:357–62. PubMed doi:10.1017/S0950268897007875

European Region

Belarus

Ivanov KS, Antonov VS, Knysh GG, Khadzhaeva AN, Antykova LP, Lavrova VP, et al. The clinical characteristics of 2 outbreaks of trichinelliasis [in Russian]. Med Parazitol (Mosk). 1990;4:41–2. PubMed

Bulgaria

Petkova S, Mihov L, Vutova K, Tsenov I, La Rosa G, Pozio E. Epidemiological and clinical patterns of trichinellosis in Bulgaria from 1995 to 2002. Parasite. 2008;15:86–8. PubMed

Croatia

Cvitović A, Miletic-Medved M, Gjenero-Margan I. An epidemic of trichinellosis in autumn 2004 in Slavonski Brod [in Croatian]. Acta Med Croatica. 2007;61:215–8. PubMed

Puljiz I, Beus A, Kuzman I, Seiwerth S. Electrocardiographic changes and myocarditis in trichinellosis: a retrospective study of 154 patients. Ann Trop Med Parasitol. 2005;99:403–11. PubMed doi:10.1179/136485905X36307

France

Ancelle T, De Bruyne A, Niang M, Poisson DM, Prazuck T, Fur A, et al. Epidémie de trichinellose à *Trichinella nativa* due à la consommation de viande d'ours, France 2005. Bulletin Épidémiologique Hebdomadaire. 2006;14:96–8.

Ancelle T, Dupouy-Camet J, Desenclos JC, Maillot E, Charlet F, Gravelat-Desclaux C, et al. Epidémie de trichinellose (France, 1993) bilan des investigations. Bulletin Épidémiologique Hebdomadaire. 1994;29:127–9.

Ancelle T, Dupouy-Camet J, Desenclos JC, Maillot E, Savage-Houze S, Drucker FCJ, et al. A multifocal outbreak of trichinellosis linked to horse meat imported from North America to France in 1993. Am J Trop Med Hyg. 1998;59:615–9. PubMed

Basset D, Thiebaut MM, Pratlong F, Abraham B, Moryoussef A, Baldet P, et al. Epidémies familiales de trichinose dues a lingestion de sanglier sauvage dans la region Languedoc. Bulletin Épidémiologique Hebdomadaire. 1995;44:195.

Bernard E, Ozouf N, Toussauint-Gari M, Marty P, Pozio E, Le Fichoux Y, et al. Deux épidémie familiales de trichinose. Med Mal Infect. 1995;25:611–4. doi:10.1016/S0399-077X(05)81038-8

Dupouy-Camet J, Ancelle T. Surveillance de la trichinellose en France. Rapport du CNR des Trichinella, 1 janvier 1999–31 decembre 2002. Rapport du CNR des Trichinella 2003.

Dupouy-Camet J, Ancelle T, Talabani H. Surveillance de la trichinellose humaine en France. Rapport du CNR des Trichinella, 1 janvier 2009–31 decembre 2009. Rapport du CNR des Trichinella 2010.

Durant J, Toussaint-Gari M, Bernard E, Marty P, Le Fichoux Y, Dellamonica P. Epidémie familiare de trichinose. Sem Hop Paris. 1991;67:1507–12.

Gaillard T, Martinaud C, Kérébel S, Cellarier G, Muzellec Y, Brisou P. A propos de deux cas de trichinellose à *Trichinella britovi.* Ann Biol Clin (Paris). 2007;65:308–12. PubMed

Gari-Toussaint M, Bernard E, Quaranta JF, Marty P, Soler C, Ozouf N, et al. First report in France of an outbreak of human trichinellosis due to Trichinella britovi. In: Campbell WC, Pozio E, Bruschi F, editors. Trichinellosis. Proceedings of the 8th International Conference on Trichinellosis, 1994. Rome: Instituto Superiore di Sanita Press; 1994. p. 465–8.

Gari-Toussaint M, Tieulié N, Baldin JL, Dupouy-Camet J, Delaunay P, Fuzibet JG, et al. Human trichinellosis due to *Trichinella britovi* in southern France after consumption of frozen wild boar meat. Euro Surveill. 2005;10:117–8. PubMed

Gari-Toussaint M, Tieulié N, Baldin JL, Marty P, Dupouy-Camet J, Delaunay P, et al. Trichinellose à *Trichinella britovi* dans les Alpes-Maritimes après consommation de viande de sanglier congelée, automne 2003. Bulletin Épidémiologique Hebdomadaire. 2004;21:87–8.

Laurichesse H, Cambon M, Perre D, Ancelle T, Mora M, Hubert B, et al. Outbreak of trichinosis in France associated with eating horse meat. Commun Dis Rep CDR Rev. 1997;7:R69–73.

Nozais JP, Mannevy V, Danis M. Deux cas de trichinose après ingestion de viande d'ours blanc (*Thalarctos maritimus*) au Groenland. Med Mal Infect. 1996;26:732–3. doi:10.1016/S0399-077X(96)80107-7

Ranque S, Faugère B, Pozio E, La Rosa G, Tamburrini A, Pelissier JF, et al. *Trichinella pseudospiralis* outbreak in France. Emerg Infect Dis. 2000;6:543–7. PubMed doi:10.3201/eid0605.000517

Roumier M, Milhe P, Hautefort B, Benoist B. Four cases of trichinosis in Camargue (France) by consumption of meat of boar [in French]. Med Mal Infect. 1992;22:947–8. doi:10.1016/S0399-077X(05)80639-0

Germany

Noeckler K, Reiter-Owona I, Heidrich J, Protz D, Rehmet S, Sinn G, et al. Aspects of clinical features, diagnosis, notification and tracing back referring to *Trichinella* outbreaks in North Rhine-Westphalia, Germany, 1998. Parasite. 2001;8:S183–5. PubMed

Nothdurft HD, Brommer M, Eichenlaub D, Löscher T. A small outbreak of trichinosis in Germany caused by imported smoked ham [in German]. Dtsch Med Wochenschr. 1995;120:173–6. PubMed doi:10.1055/s-2008-1055330

Rehmet S, Sinn G, Robstad O, David H, Lesser D, Noeckler K, et al. Two outbreaks of trichinellosis in the state of Northrhine-Westfalia, Germany, 1998. Euro Surveill. 1999;4:78–81. PubMed

Robert Koch Institute. InstitutSurStat@RKI, 2010. Human cases of trichinellosis in Germany from 2001 to 12 May 2010.

Israel

Marva E, Markovics A, Gdalevich M, Asor N, Sadik C, Leventhal A. Trichinellosis outbreak. Emerg Infect Dis. 2005;11:1979–81. PubMed

Italy

Frongillo RF, Baldelli B, Pozio E, Crapa G, Di Giuli C, Santirocchi M, et al. Report on an out break of trichinellosis in central Italy. Eur J Epidemiol. 1992;8:283–8. PubMed doi:10.1007/BF00144815

Pagni P, Bindi M, Burzagli L, Melchior M. An outbreak of trichinellosis in central Italy caused by pork meat ingestion. In: Campbell WC, Pozio E, Bruschi F, editors. Trichinellosis. Proceedings of the 8th International Conference on Trichinellosis, 1994. Rome: Instituto Superiore di Sanita Press; 1994. p. 487–90.

Pozio E, Cappelli O, Marchesi L, Valeri P, Rossi P. Third outbreak of trichinellosis caused by consumption of horse meat in Italy. Ann Parasitol Hum Comp. 1988;63:48–53.

Pozio E, Santagada G, Di Bari C. Outbreak of trichinellosis in southern Italy. Trans R Soc Trop Med Hyg. 1986;80:997–8. PubMed doi:10.1016/0035-9203(86)90291-9

Pozio E, Varese P, Gomez Morales MA, Croppo GP, Pelliccia D, Bruschi F. Comparison of human trichinellosis caused by *Trichinella spiralis* and *Trichinella britovi.* Am J Trop Med Hyg. 1993;48:568–75. PubMed

Tamburrini A, Sacchini D, Pozio E. An expected out break of human trichinellosis for the consumption of horsemeat. Parasite. 2001;8:S186–7. PubMed

Lithuania

Bartuliene A, Liausediene R, Motiejuniene V. Trichinellosis outbreak in Lithuania, Ukmerge region, June 2009. Euro Surveill. 2009;14:pii: 19336. PubMed

Romania

Neghina R, Neghina AM, Marincu I, Moldovan R, Iacobiciu I. Epidemiological and diagnostic findings during a 16-year-long trichinellosis surveillance in Timis County, Romania. Vet Parasitol. 2009;159:328–31. PubMed doi:10.1016/j.vetpar.2008.10.045

Neghina R, Neghina AM, Marincu I, Moldovan R, Iacobiciu I. Trichinellosis, a threatening and re-emerging disease in a Romanian western county. Vector Borne Zoonotic Dis. 2009;9:717–21. PubMed doi:10.1089/vbz.2008.0204

Slovakia

Paraličová Z, Kincekova J, Schreter I, Jarcuska P, Dubinsky P, Porubcin S, et al. Outbreak of trichinellosis in eastern Slovakia. Helminthologia. 2009;46:209–13. doi:10.2478/s11687-009-0039-2

Reiterová K, Kincekova J, Snabel V, Marucci G, Pozio E, Dubinsky P. *Trichinella spiralis*–outbreak in the Slovak Republic. Infection. 2007;35:89–93. PubMed doi:10.1007/s15010-007-6122-z

Spain

Arévalo Velasco A, Bringas MJ, Rodriguez R, Menor A. Description de un brote de triquinosis en la provincia de Salamanca. Rev Esp Quimioter. 2009;22:115–6. PubMed

Benitez JA, Cruz JM, Adrados G, Gonzalez T. Triquinosis: a proposito de 17 casos aparecidos en un mismo brote. Enferm Infecc Microbiol Clin. 1987;5:122–3.

Cobo J, Gómez Cerezo J, Medraño JC, Zapico R, Cruz Martinez A, Molina F, et al. Trichinosis. A study of a large outbreak on the Iberian peninsula [in Spanish]. An Med Interna. 1991;8:441–4. PubMed

de la Cruz de Julián I, Diaz Garcia JM, Alvarez Lana P, Garcia Colmenero C. An outbreak of trichinosis in Huerta del Marquesado (the Cañete-Cuenca basic health area). December 1992 to January 1993 [in Spanish]. Rev Sanid Hig Publica (Madr). 1994;68:513–20. PubMed

de la Torre Cecilia C, Espino Aguilar R, Cardenas Talaveron C, Caňuelo Ruiz O, Garrido Palomo R, Baena Saez J, et al. Triquinosis: asportacion de dos casos. An Esp Pediatr. 1989;30:227–8. PubMed

Perucha González M, Lezaun Larumbe ME, Torres Baile JL, Campo Hernández JM, Bernal Martínez A. Outbreak of trichinosis in various localities of Rioja Baja [in Spanish]. Rev Sanid Hig Publica (Madr). 1987;61:1035–47. PubMed

Herráez García J, Leon Garcia LA, Lanusse Senderos C, Cortés Blanco M, García Cabanas A. Outbreak of trichinellosis in the region of la Vera (Carceres, Spain) caused by *Trichinella britovi* [in Spanish]. An Med Interna. 2003;20:63–6. PubMed doi:10.4321/S0212-71992003000200003

López Hernández B, Gea Velazquez de Castro MT, Galicia Garcia MD, Sabonet JC. Brote epidemico por *Trichinella britovi* en Granada durante la primavera del 2000. Rev Esp Salud Publica. 2001;75:467–73. PubMed doi:10.1590/S1135-57272001000500007

Mangas Gallardo I, Tello Anchuela O. Vigilancia de la triquinosis en Espana. Temporadas 1989/90–1992/93. Bol. Epidemiol. Microbiol. 1994;1:233–4.

Martinez Corral JJ, Esteban Niveiro MJ, Oliet Pala R. Triquinosis producida por el consumo de carne de porcino infestado con *Trichinella spiralis*. Investigation y estudio de un brote alimentario. Salud Publica. 2000;12:36–40.

Serrano R, Lacasa J, Velazquez J, Ziad F, Aznar R. Triquinosis: nuevo brote epidémico por ingesta de embutido de jabali. Enferm Infecc Microbiol Clin. 1989;7:428–31. PubMed

Tiberio G, Rivero M, Lanzas G, Redin D, Ardanaz E, Fernandez C, et al. Triquinelosis: estudio de dos brotes en Navarra. Enferm Infecc Microbiol Clin. 1997;15:151–3. PubMed

Turkey

Akkoc N, Kuruuzum Z, Aker S, Yuce A, Onen F, Yapar N, et al. A large-scale outbreak of trichinellosis caused by *Trichinella britovi* in Turkey. Zoonoses Public Health. 2009;56:65–70. PubMed doi:10.1111/j.1863-2378.2008.01158.x

Heper Y, Yilmaztepe F, Komitova R, Akalin H, Vutova K, Helvaci S. A trichinosis outbreak caused by wild boar meat in Turkey. Parasite. 2005;12:191–2. PubMed

United Kingdom

Milne LM, Bhagani S, Bannister BA, Laitner SM, Moore P, Eza D, et al. Trichinellosis acquired in the United Kingdom. Epidemiol Infect. 2001;127:359–63. PubMed doi:10.1017/S0950268801005994

Western Pacific Region

China

Cui J, Wang ZQ, Wu F, Jin XX. Epidemiological and clinical studies on an outbreak of trichinosis in central China. Ann Trop Med Parasitol. 1997;91:481–8. PubMed doi:10.1080/00034989760842

Gong XH, Guo WM, Cirendunzhu, Long EK, Ma Y, Bianbazuoma, et al. Investigation on an outbreak of trichinosis and clinical analysis [in Chinese]. Zhongguo Ji Sheng Chong Xue Yu Ji Sheng Chong Bing Za Zhi. 2008;26:79–80. PubMed

Lo YC, Hung CC, Lai CS, Wu Z, Nagano I, Maeda T, et al. Human trichinosis after consumption of soft-shelled turtles, Taiwan. Emerg Infect Dis. 2009;15:2056–8. PubMed doi:10.3201/eid1512.090619

Luo Z, Li W, Shen L. Report of an outbreak of trichinellosis. Chin J Zoonoses. 2004;20:359–60.

Wang Z, Cai J, Wa F, Jin X. Seven outbreaks of trichinosis in China (1992–1996). J Egypt Soc Parasitol. 1997;27:529–38. PubMed

Xu B, Chang J, Gao G, Tang B, Hong J, Chen Y, et al. Four outbreaks of human trichinellosis in Henan Province. Chin Med J (Engl). 1995;108:872–4. PubMed

Japan

Nakamura T, Miura T, Nakaoka T, Nagano I, Takahashi Y, Iwamoto A. A case of trichinellosis with spontaneous remission. J J A Inf Dis. 2003;77:839–43.

Shiota T, Arizono N, Yoshioka T, Ishikawa Y, Fujitake J, Fujii H, et al. Imported trichinellosis with severe myositis—report of a case [in Japanese]. Kansenshogaku Zasshi. 1999;73:76–82. PubMed

Korea

Kim E, Pyun RH, Park JH, Kim KH, Choi I, Park HH, et al. Family outbreak of trichinosis after eating a raw meat of wild swine. Korean J Parasitol. 2003;35:180–4.

Lee HC, Kim HJ, Oh HY, Kim JH, Kim HG, Lee MS, et al. A case of trichinosis caused by eating a wild badger. Korean J Med. 1999;56:134–8.

Sohn WM, Huh S, Chung DI, Pozio E. Molecular identification of Korean *Trichinella* isolates. Korean J Parasitol. 2003;41:125–7. PubMed doi:10.3347/kjp.2003.41.2.125

Singapore

Kurup A, Yew WS, San LM, Ang B, Lim S, Tai GK. Outbreak of suspected trichinosis among travelers returning from a neighboring island. J Travel Med. 2000;7:189–93. PubMed doi:10.2310/7060.2000.00058

Laos

Barennes H, Sayasone S, Odermatt P, De Bruyne A, Hongsakhone S, Newton PN, et al. A major trichinellosis outbreak suggesting a high endemicity of *Trichinella* infection in northern Laos. Am J Trop Med Hyg. 2008;78:40–4. PubMed

Sayasone S, Odermatt P, Vongphrachanh P, Keoluangkot V, Dupouy-Camet J, Newton PN, et al. A trichinellosis outbreak in Borikhamxay Province, Lao PDR. Trans R Soc Trop Med Hyg. 2006;100:1126–9. PubMed doi:10.1016/j.trstmh.2006.01.010

Vietnam

De NV, Toan ND, Son DT, Van P. Report an epidemic area of trichinellosis in Tuan Giao district, Lai Chau province. Proc 5th Seminar on Food- and Water-Borne Parasitic Zoonoses. Bangkok, Thailand. 2006 Nov 28–30; p. 48.

Taylor WRJ, Tran GV, Nguyen TQ, Dang DV, Nguyen VK, Nguyen CT, et al. Acute febrile myalgia in Vietnam due to trichinellosis following the consumption of raw pork. Clin Infect Dis. 2009;49:e79–83. PubMed doi:10.1086/605533

South-East Asia Region

Thailand

Jongwutiwes S, Chantachum N, Kraivichian P, Siriyasatien P, Putaporntip C, Tamburrini A, et al. First outbreak of human trichinellosis caused by *Trichinella pseudospiralis*. Clin Infect Dis. 1998;26:111–5. PubMed doi:10.1086/516278

Khumjui C, Choomkasien P, Dekumyoy P, Kusolsuk T, Kongkaew W, Chalamaat M, et al. Outbreak of trichinellosis caused by *Trichinella papuae*, Thailand. 2006. Emerg Infect Dis. 2008;14:1913–5. PubMed doi:10.3201/eid1412.080800

Kusolsuk T, Kamonrattanakun S, Wesanonthawech A, Dekumyoy P, Thaenkham U, Yoonuan T, et al. The second outbreak of trichinellosis caused by *Trichinella papuae* in Thailand. Trans R Soc Trop Med Hyg. 2010;104:433–7. PubMed doi:10.1016/j.trstmh.2009.12.005

Limsuwan S, Siriprasert V. A clinical study on trichinosis in Changwat Phayao, Thailand. Southeast Asian J Trop Med Public Health. 1994;25:305–8. PubMed

Watt G, Saisorn S, Jongsakul K, Sakolvaree Y, Chaicumpa W. Blinded, placebo-controlled trial of antiparasitic drugs for trichinosis myositis. J Infect Dis. 2000;182:371–4. PubMed doi:10.1086/315645

India

Alipuria S, Sangha HK, Singh G, Pandhi S. Trichinosis—a case report. Indian J Pathol Microbiol. 1996;39:231–2. PubMed

Handa R, Aggarwal P, Sarkar C, Vijayarghavan M, Mattewal A, Arya V, et al. A patient with muscle weakness. J Indian Rheum Assoc. 2000;8:85–7.

Section D. Summary of sex and age data on persons with trichinellosis cases, by World Health Organization region, 1986 to 2009

Region/Country	% Males (total no. cases)*	Age, y, of infected persons (no. cases)	References
African Region			
Ethiopia	100 (28)	Range: 23–25 (3)<br>Mean: 24 (3)	Kefenie et al., 1988<br>Kefenie and Bero, 1992
Region of the Americas			
Canada	62.1 (150)	Range: 21–67 (85)<br>Mean: 34.4 (65)	MacLean et al., 1992<br>Greenbloom et al., 1997<br>Schellenberger et al., 2003<br>Serhir et al., 2001<br>Ancelle et al., 2006
USA	57.5 (632)	Range: 1–87 (412)<br>Mean: 42.0 (126)<br>Median: 37.1 (232)	McAuley et al. 1991<br>McAuley et al., 1992<br>Dworkin et al., 1996<br>Moorhead et al., 1999<br>Roy et al., 2003<br>Kennedy et al., 2009
Mexico	35 (59)	Range: 25–44 (59)	Hernández et al., 1992
Chile	60 (667)	Range: 5–70 (667)	Zamorano et al., 1994<br>Schenone et al., 2002
Eastern Mediterranean Region			
Lebanon	54 (44)	Range: 10–70 (44)<br>Mean: 33 (44)	Haim et al., 1997
European Region			
Bulgaria	49 (228)	Range: 1–70 (228)	Petkova et al., 2008
Croatia	57 (200)	Range: 3–67 (200)<br>Mean: 35 (200)	Cvitovic et al., 2007<br>Venus et al., 2008
Czech Republic	41.9 (31)	Range: 9–68 (31)<br>Mean: 35.9 (31)	National Reference Laboratory for Tissue Helminthosis
France	51.4 (586)	Range: 1–84 (581)<br>Mean: 43.8 (581)	Durant et al., 1991<br>Roumier et al., 1992<br>Laurichesse et al., 1997<br>Bernard et al., 1995<br>Ancelle et al., 1998<br>Ranque et al., 2000<br>Gari-Toussaint et al., 2004<br>Ancelle et al., 2006<br>Gaillard et al., 2007<br>Dupouy-Camet et al., 2003<br>Dupouy-Camet et al., 2005<br>Dupouy-Camet et al., 2010
Germany	51.9 (104)	Range: 1–73 (101)<br>Mean: 34.8 (101)	Jansen et al., 2008<br>Nothdurft et al., 1995<br>Schmiedel and Kramme, 2007<br>Nockler et al., 2007
Israel	100 (26)	Mean 32 (26)	Marva et al., 2005
Italy	50.3 (382)	Range: 1–90 (368)<br>Mean: 36.7 (368)	Pozio et al., 1986<br>Pozio et al., 1988<br>Frongillo et al., 1992<br>Pozio et al., 1993<br>Tamburrini et al., 2001
Romania	53.2 (521)	Range: 1– >60 (521)<br>Mean: 31.4 (521)	Neghina et al., 2009
Slovakia	63.6 (11)	Range: 16–80 (21)<br>Mean: 40.5 (21)	Reiterová et al., 2007<br>Paraličová et al., 2009

Spain	57.5 (237)	Range: 2–86 (140)<br>Mean: 40.7 (177)	Benitez et al., 1987<br>Serrano et al., 1989<br>Cobo et al., 1991<br>de la Cruz de Julián et al., 1994<br>Rodriguez-Osorio et al., 1999<br>López Hernández et al., 2001<br>Cortés Blanco et al., 2002<br>Herráez Garcia et al., 2003
Turkey	52.6 (418)	Range: 1.5–73 (418)<br>Mean: 31.1 (418)	Akkoc et al., 2009
South-East Asia Region			
Thailand	71 (165)	Range: 7–70 (210)<br>Mean: 35.6 (208)<br>Median: 34.5 (140)	Limsuwan and Siripraesert, 1994<br>Jongwutiwes et al., 1998<br>Charkrit, 1998<br>Khumjui et al., 2008<br>Kusolsuk et al., 2010
Western Pacific Region			
China	58.2 (802)	Range: 1–90 (482)	Xu et al., 1995<br>Cui et al., 1997<br>Wang et al., 1997<br>Ye et al., 2003<br>Luo et al., 2004
Laos	47% (111)	Range: 5–69 (111)<br>Median: 34 (21)<br>Mean: 30.4 (90)	Sayasone et al., 2006<br>Barennes et al., 2008
Singapore	56% (25)	Mean: 22.5 (25)	Kurup et al., 2000
Vietnam	92% (42)	Range: 20–60 (42)<br>Mean: 45.4 (42)	De at al., 2000

*Data are from those reports which presented adequate sex and age data of no fewer than 10 cases during 1986–2009.

References

African Region

Ethiopia

Kefenie H, Bero G. Trichinosis from wild boar meat in Gojjam, north-west Ethiopia. Trop Geogr Med. 1992;44:278–80. PubMed

Kefenie H, Wolde H, Abuherpo M. Trichinosis from wild boar meat in Arsi, central Ethiopia. Ethiop Med J. 1988;26:97–100. PubMed

Region of the Americas

Canada

Ancelle T, Bruyne A, Nuang M, Poisson DM, Prazuck T, Fur A, et al. Epidemic of *Trichinella nativa* trichinosis caused by consumption of bear meat, France, 2005. Bulletin Épidémiologique Hebdomadaire. 2006;14:96–8.

Greenbloom SL, Martin-Smith P, Issacs S, Marshall B, Kittle DC, Kain KC, et al. Outbreak of trichinosis in Ontario secondary to the ingestion of wild boar meat. Can J Public Health. 1997;88:52–6. PubMed

MacLean JD, Poirier L, Gyorkos TW, Proulx JF, Bourgeault L, Corriveau A, et al. Epidemiologic and serologic definition of primary and secondary trichinosis in the Arctic. J Infect Dis. 1992;165:908–12. PubMed

Schellenberg RS, Tan B, Irvine JD, Stockdale DR, Gajadhar AA, Serhir B, et al. An outbreak of trichinellosis due to consumption of bear meat infected with *Trichinella nativa* in two northern Saskatchewan communities. J Infect Dis. 2003;188:835–43. PubMed

Serhir B, MacLean JD, Healey S, Forbes L. Outbreak of trichinellosis associated with arctic walruses in northern Canada, 1999. Can Commun Dis Rep. 2001; 27–04:31–6.

United States

Dworkin MS, Gamble HR, Zarlenga DS, Tennican PO. Outbreak of trichinellosis associated with eating cougar jerky. J Infect Dis. 1996;174:663–6. PubMed

Kennedy ED, Hall RL, Montgomery SP, Pyburn DG, Jones JJ. Trichinellosis surveillance—United States, 2002–2007. MMWR Surveill Summ. 2009;58(9):1–7. PubMed

McAuley JB, Michelson MK, Schantz PM. Trichinellosis surveillance, United States, 1987–1990. MMWR CDC Surveill Summ. 1991;40(3):35–42. PubMed

McAuley JB, Michelson MK, Hightower AW, Engeran S, Wintermeyer LA, Schantz PM. A trichinosis outbreak among Southeast Asian refugees. Am J Epidemiol. 1992;135:1404–10. PubMed

Moorhead A, Grunenwald PE, Dietz VJ, Schantz PM. Trichinellosis in the United States, 1991–1996: declining but not gone. Am J Trop Med Hyg. 1999;60:66–9. PubMed

Roy SL, Lopez AS, Schantz PM. Trichinellosis surveillance—United States, 1997–2001. MMWR Surveill Summ. 2003;52(6):1–8. PubMed

Mexico

Hernández M, Ramos-Martinez E, Casco-Sánchez EJ, Morales-Gómez JM, Pérez-Matos EM. Acute trichinosis. Epidemics of 166 cases in Delicias City, Chih. Diagnosis with tissue compression and staining [in Spanish]. Gac Med Mex. 1992;128:45–50. PubMed

Chile

Schenone H, Olea A, Schenone H, Contreceras MC, Mercado R, Sandoval L, et al. Epidemiology of trichinosis in Chile from 1991–2000. Rev Med Chil. 2002;130:281–5. PubMed

Zamorano CG, Contreras MC, Espinoza A, Paredes M, Sandoval L, Schulz E, et al. Brote de triquinosis en la Comuna de Purranque, X Region, Chile, Octubre–Noviembre, 1992. Bol Chil Parasitol. 1994;49:38–42. PubMed

Eastern Mediterranean Region

Lebanon

Haim M, Efrat M, Wilson M, Schantz PM, Cohen D, Shemer J. An outbreak of *Trichinella spiralis* infection in southern Lebanon. Epidemiol Infect. 1997;119:357–62. PubMed

European Region

Bulgaria

Petkova S, Mihov L, Vutova K, Tsenov I, La Rosa G, Pozio E. Epidemiological and clinical patterns of trichinellosis in Bulgaria from 1995 to 2002. Parasite. 2008;15:86–8. PubMed

Croatia

Cvitović A, Miletić-Medved M, Gjenero-Margan I. An epidemic of trichinellosis in autumn 2004 in Slavonski Brod [in Croatian]. Acta Med Croatica. 2007;61:215–8. PubMed

Venus M, Puntaric D, Grgic M, Gmajnic R, Miskulin M. The effect of pest control on the incidence of trichinosis in Virovitica-Podravina county, Croatia. Vet Parasitol. 2008;156:226–33.

Czech Republic

National Reference Laboratory for Tissue Helminthosis of the Czech Republic 1986–2009 [email: Libuse.kolarova@lfl.cuni.cz]

France

Ancelle T, Dupouy-Camet J, Desenclos JC, Maillot E, Savage-Houze S, Drucker FCJ, et al. A multifocal outbreak of trichinellosis linked to horse meat imported from North America to France in 1993. Am J Trop Med Hyg. 1998;59:615–9. PubMed

Ancelle T, De Bruyne A, Niang M, Poisson DM, Prazuck T, Fur A, et al. Epidémie de trichinellose à *Trichinella nativa* due à la consommation de viande d’ours, France 2005. Bulletin Épidémiologique Hebdomadaire. 2006;14:96–8.

Bernard E, Ozouf N, Toussauint-Gari M, Marty P, Pozio E, Le Fichoux Y, et al. Deux épidémie familiales de trichinose. Med Mal Infect. 1995;25:611–4.

Dupouy-Camet J, Ancelle T. Surveillance de la trichinellose en France. Rapport du CNR des Trichinella, 1 janvier 1999–31 decembre 2002. Rapport du CNR des Trichinella 2003.

Dupouy-Camet J, Ancelle T, de Bruyne A. Surveillance de la trichinellose humaine en France. Rapport du CNR des Trichinella, 1 janvier 2002–31 decembre 2004. Rapport du CNR des Trichinella 2005.

Dupouy-Camet J, Ancelle T, Talabani H. Surveillance de la trichinellose humaine en France. Rapport du CNR des Trichinella, 1 janvier 2009–31 decembre 2009. Rapport du CNR des Trichinella 2010.

Durant J, Toussaint-Gari M, Bernard E, Marty P, Le Fichoux Y, Dellamonica P. Epidémie familiare de trichinose. Sem Hop Paris. 1991;67:1507–12.

Gaillard T, Martinaud C, Kérébel S, Cellarier G, Muzellec Y, Brisou P. A propos de deux cas de trichinellose à *Trichinella britovi.* Ann Biol Clin (Paris). 2007;65:308–12. PubMed

Gari-Toussaint M, Tieulié N, Baldin JL, Marty P, Dupouy-Camet J, Delaunay P, et al. Trichinellose à *Trichinella britovi* dans les Alpes-Maritimes après consommation de viande de sanglier congelée, automne 2003. Bulletin Épidémiologique Hebdomadaire. 2004;21:87–8.

Laurichesse H, Cambon M, Perre D, Ancelle T, Mora M, Hubert B, et al. Outbreak of trichinosis in France associated with eating horse meat. Commun Dis Rep CDR Rev. 1997;7:R69–73.

Ranque S, Faugère B, Pozio E, La Rosa G, Tamburrini A, Pelissier JF, et al. *Trichinella pseudospiralis* outbreak in France. Emerg Infect Dis. 2000;6:543–7. PubMed

Roumier M, Milhe P, Hautefort B, Benoist B. Four cases of trichinosis in Camargue (France) by consumption of meat of boar [in French]. Med Mal Infect. 1992;22:947–8. doi:10.1016/S0399-077X(05)80639-0

Germany

Jansen A, Schöneberg I, Stark K, Nöckler K. Epidemiology of trichinellosis in Germany, 1996–2006. Vector Borne Zoonotic Dis. 2008;8:189–96. PubMed

Noeckler K, Reiter-Owona I, Heidrich J, Protz D, Rehmet S, Sinn G, et al. Aspects of clinical features, diagnosis, notification and tracing back referring to *Trichinella* outbreaks in North Rhine-Westphalia, Germany, 1998. Parasite. 2001;8:S183–5. PubMed

Nöckler K, Wichmann-Schauer H, Hiller P, Müller A, Bogner K. Trichinellosis outbreak in Bavaria caused by cured sausages from Romania, January 2007. Euro Surveill. 2007; 12:E070823.2. PubMed

Nothdurft HD, Brommer M, Eichenlaub D, Löscher T. A small outbreak of trichinosis in Germany caused by imported smoked ham [in German]. Dtsch Med Wochenschr. 1995;120:173–6. PubMed

Schmiedel S, Kramme S. Cluster of trichinellosis cases in Germany, imported from Poland, June 2007. Euro Surveill. 2007;12:E070719.4. PubMed

Israel

Marva E, Markovics A, Gdalevich M, Asor N, Sadik C, Leventhal A. Trichinellosis outbreak. Emerg Infect Dis. 2005;11:1979–81. PubMed

Italy

Frongillo RF, Baldelli B, Pozio E, Crapa G, Di Giuli C, Santirocchi M, et al. Report on an outbreak of trichinellosis in central Italy. Eur J Epidemiol. 1992;8:283–8. PubMed

Pozio E, Santagada G, Di Bari C. Outbreak of trichinellosis in southern Italy. Trans R Soc Trop Med Hyg. 1986;80:997–8. PubMed

Pozio E, Cappelli O, Marchesi L, Valeri P, Rossi P. Third outbreak of trichinellosis caused by consumption of horse meat in Italy. Ann Parasitol Hum Comp. 1988;63:48–53.

Pozio E, Varese P, Gomez Morales MA, Croppo GP, Pelliccia D, Bruschi F. Comparison of human trichinellosis caused by *Trichinella spiralis* and *Trichinella britovi.* Am J Trop Med Hyg. 1993;48:568–75. PubMed

Tamburrini A, Sacchini D, Pozio E. An expected out break of human trichinellosis for the consumption of horsemeat. Parasite. 2001;8:S186–7. PubMed

Romania

Neghina R, Neghina AM, Marincu I, Moldovan R, Iacobiciu I. Epidemiological and diagnostic findings during a 16-year-long trichinellosis surveillance in Timis County, Romania. Vet Parasitol. 2009;159:328–31. PubMed

Slovakia

Paraličová Z, Kinceková J, Schreter I, Jarcuska P, Dubinsky P, Porubcin S, et al. Outbreak of trichinellosis in eastern Slovakia. Helminthologia. 2009;46:209–13.

Reiterová K, Kinceková J, Snábel V, Marucci G, Pozio E, Dubinsky P. *Trichinella spiralis*–outbreak in the Slovak Republic. Infection. 2007;35:89–93. PubMed

Spain

Benitez JA, Cruz JM, Adrados G, Gonzalez T. Triquinosis: a proposito de 17 casos aparecidos en un mismo brote. Enferm Infecc Microbiol Clin. 1987;5:122–3.

Cobo J, Gómez Cerezo J, Medraño JC, Zapico R, Cruz Martinez A, Molina F, et al. Trichinosis. A study of a large outbreak on the Iberian peninsula [in Spanish]. An Med Interna. 1991;8:441–4.

Cortés Blanco M, Garciá-Cabañas A, Guerra-Peguero F, Ramos-Aceitero JM, Herrera-Guibert D, Martínez-Navarro JF. Outbreak of trichinellosis in Cáceres, Spain, December 2001–February 2002. Euro Surveill. 2002;7:136–8 PubMed

de la Cruz de Julián I, Diaz Garcia JM, Alvarez Lana P, Garcia Colmenero C. An outbreak of trichinosis in Huerta del Marquesado (the Cañete-Cuenca basic health area). December 1992 to January 1993 [in Spanish]. Rev Sanid Hig Publica (Madr). 1994;68:513–20.

Herráez García J, Leon García LA, Lanusse Senderos C, Cortés Blanco M, García Cabañas A. Brote de triquinosis en la comarca de la Vera (Caceres) causado por *Trichinella britovi.* An Med Interna. 2003;20:63–6. PubMed

López Hernández B, Velázquez de Castro MT, Galicia García MD, Sabonet JC. Brote epidemico por *Trichinella britovi* en Granada durante la primavera del 2000. Rev Esp Salud Publica. 2001;75:467–73. PubMed

Rodriguez-Osorio M, Abad JM, De Haro T, Villa-Real R, Gomez Garcia V. Human trichinellosis in southern Spain: serologic and epidemiologic study. Am J Trop Med Hyg. 1999;61:834–7. PubMed

Serrano R, Lacasa J, Velazquez J, Ziad F, Aznar R. Triquinosis: nuevo brote epidémico por ingesta de embutido de jabali. Enferm Infecc Microbiol Clin. 1989;7:428–31. PubMed

Turkey

Akkoc N, Kuruuzum Z, Aker S, Yuce A, Onen F, Yapar N, et al. A large-scale out break of trichinellosis caused by *Trichinella britovi* in Turkey. Zoonoses Public Health. 2009;56:65–70. PubMed

South-East Asia Region

Thailand

Charkrit S. Study on clinical manifestations of trichinosis in Payao province. Communicable Diseases Journal. 1998;24:242–7.

Limsuwan S, Siriprasert V. A clinical study on trichinosis in Changwat Phayao, Thailand. Southeast Asian J Trop Med Public Health. 1994;25:305–8. PubMed

Jongwutiwes S, Chantachum N, Kraivichian P, Siriyasatien P, Putaporntip C, Tamburrini A, et al. First outbreak of human trichinellosis caused by *Trichinella pseudospiralis.* Clin Infect Dis. 1998;26:111–5. PubMed

Khumjui C, Choomkasien P, Dekumyoy P, Kusolsuk T, Kongkaew W, Chalamaat M, et al. Outbreak of trichinellosis caused by *Trichinella papuae*, Thailand. 2006. Emerg Infect Dis. 2008;14:1913–5. PubMed

Kusolsuk T, Kamonrattanakun S, Wesanonthawech A, Dekumyoy P, Thaenkham U, Yoonuan T, et al. The second outbreak of trichinellosis caused by *Trichinella papuae* in Thailand. Trans R Soc Trop Med Hyg. 2010;104:433–7. PubMed

Western Pacific Region

China

Cui J, Wang ZQ, Wu F, Jin XX. Epidemiological and clinical studies on an outbreak of trichinosis in central China. Ann Trop Med Parasitol. 1997;91:481–8. PubMed

Luo Z, Li W, Shen L. Report of an outbreak of trichinellosis. Chinese Journal of Zoonoses. 2004;20:359–60.

Wang Z, Cui J, Wu F, Jin X. Seven outbreaks of trichinosis in China (1992–1996). J Egypt Soc Parasitol. 1997;27:529–38. PubMed

Xu B, Chang J, Gao G, Tang B, Hong J, Chen Y, et al. Four outbreaks of human trichinellosis in Henan Province. Chin Med J (Engl). 1995;108:872–4. PubMed

Ye P, Jianhua H, Zhengkun Z, Desong G, Hao W. The investigation over an outbreak of trichinellosis caused by eating *Sus scrofa.* Chinese Journal of Zoonoses. 2003;19:12.

Laos

Barennes H, Sayasone S, Odermatt P, De Bruyne A, Hongsakhone S, Newton PN, et al. A major trichinellosis outbreak suggesting a high endemicity of *Trichinella* infection in northern Laos. Am J Trop Med Hyg. 2008;78:40–4. PubMed

Sayasone S, Odermatt P, Vongphrachanh P, Keoluangkot V, Dupouy-Camet J, Newton PN, et al. A trichinellosis outbreak in Borikhamxay Province, Lao PDR. Trans R Soc Trop Med Hyg. 2006;100:1126–9. PubMed

Singapore

Kurup A, Yew WS, San LM, Ang B, Lim S, Tai GK. Outbreak of suspected trichinosis among travelers returning from a neighboring island. J Travel Med. 2000;7:189–93. PubMed

Vietnam

De NV, Toan ND, Son DT, Van P. Report an epidemic area of trichinellosis in Tuan Giao district, Lai Chau province. Proc 5th Seminar on Food- and Water-Borne Parasitic Zoonoses. Bangkok, Thailand. 2006 Nov 28–30. p. 48.
