# Supplementary material for: Worldwide Occurrence and Impact of Human Trichinellosis, 1986–2009
Source: Emerg Infect Dis. 2011 Dec;17(12):2194–202. doi: 10.3201/eid1712.110896 (PMC3311199; doi:10.3201/eid1712.110896)
Supplement: Related texts

# Review article: Worldwide Occurrence and Impact of Human Trichinellosis, 1986–2009

**DOI:** 10.3201/eid1712.110896

**Authors:** K. Darwin Murrell, Edoardo Pozio

**Affiliations:** Author affiliations: University of Copenhagen, Copenhagen, Denmark (K.D. Murrell);; Istituto Superiore di Sanità, Rome, Italy (E. Pozio)

## Abstract

To assess the global incidence and clinical effects of human trichinellosis, we analyzed outbreak report data for 1986–2009. Searches of 6 international databases yielded 494 reports. After applying strict criteria for relevance and reliability, we selected 261 reports for data extraction. From 1986 through 2009, there were 65,818 cases and 42 deaths reported from 41 countries. The World Health Organization European Region accounted for 87% of cases; 50% of those occurred in Romania, mainly during 1990–1999. Incidence in the region ranged from 1.1 to 8.5 cases per 100,000 population. Trichinellosis affected primarily adults (median age 33.1 years) and about equally affected men (51%) and women. Major clinical effects, according to 5,377 well-described cases, were myalgia, diarrhea, fever, facial edema, and headaches. Pork was the major source of infection; wild game sources were also frequently reported. These data will be valuable for estimating the illness worldwide.

Since the mid-19th century, trichinellosis has been a well-recognized meat-borne zoonosis; however, despite concerted control efforts, it remains a threat in many countries. Veterinary control over the slaughter of food animals to ensure food safety, particularly meat inspection, was introduced in Germany in 1866 specifically to prevent trichinellosis from pork infected with the muscle larvae of *Trichinella spiralis* (*1*). In the European Union, the estimated annual cost incurred from meat inspection of 167 million pigs (*2*) ranges from €25 million to €400 million (*3*). Even in countries without mandatory meat inspection (e.g., United States), the economic cost of selling pork in international and national markets is substantial (*4*).

The epidemiology and systematics (i.e., the study of the diversification) of this zoonosis are now recognized to involve, in addition to *T. spiralis*, 7 other species in 4 genotypes, all of which are more commonly found in wild animals than in domestic pigs (*5*). *Trichinella* spp. have been found in domestic and wild animals in 66 countries (*6*). Human trichinellosis has been documented in 55 countries, particularly those with well-established food behavior that includes consuming meat dishes with raw or undercooked meat (*6*). Whether trichinellosis is a low-prevalence disease or is frequently misdiagnosed is not clearly understood; its detection can be difficult in low-level infections and its clinical manifestations overlap those of other diseases, such as influenza and chronic fatigue syndrome (*7*). Human infection is classically characterized by gastroenteritis; myalgia; malaise; facial edema; headache; subungual or conjunctival hemorrhages; and increased eosinophils, leukocytes, and muscle enzymes (*7*).

Reliable estimates of the incidence of trichinellosis among humans and its effect on health are not available; these estimates are necessary for setting priorities. In the 1990s, the global prevalence of trichinellosis was ≈10 million, and a recent incidence estimate suggested ≈10,000 infections per year (*6*). However, because of problems related to incomplete data from some regions and to the quality of diagnostic criteria for infection, the Foodborne Disease Burden Epidemiology Reference Group of the World Health Organization (WHO) requested a systematic review of the global incidence, burden of disease, and major sources of infection that used strict criteria for data selection and extraction. Our analyses and summaries of the epidemiologic and clinical data selected provide a basis for an assessment of trichinellosis as a public health problem.

## Data Sources and Selection Criteria

We retrospectively reviewed trichinellosis outbreak investigations conducted worldwide during 1986–2009. The data analysis focused on incidence, age and sex of patients, infection rates, major clinical aspects (including sequelae), and meat sources of infection. The database we developed was geographically organized according to the WHO regions (www.who.int/choice/demography/regions/en): African Region, 46 countries; Region of the Americas, 12 countries; Eastern Mediterranean Region, 22 north African and Middle Eastern countries; European Region, 44 European and 6 Asian countries; South-East Asian Region, 11 east Asian countries; and Western Pacific Region, 27 countries. Data searches of literature included PubMed, Centres for Agricultural Bioscience International (CABI) abstracts, WHO library, System for Information on Gray Literature, Pan America Health Organization Virtual Library, and Index Medicus for South-East Asian Region.

The search terms used were trichinosis, trichinellosis, trichinelliasis, and trichinella. These were combined with the terms prevalence, outbreaks, epidemiology, clinical symptoms, and duration. The search terms were also combined with pork, pig, wild boar, wild pig, warthog, horse, badger, jackal, cougar, walrus, armadillo, turtle, and bear meat. Published abstracts were screened for retention by using the criteria of relevance to human outbreaks or single cases occurring from 1986 through 2009 and by determining whether the report was based on original data (primary source or unpublished data managed by national government agencies). The full paper versions of selected abstracts were then obtained where possible and further screened and evaluated. Outbreak reports published >1 time were occasionally encountered, and care was taken to prevent duplication of data in the database; preference was given to published international, peer-reviewed versions. In some instances, data were obtained through contact with scientists in countries of interest who had access to unpublished and detailed information about outbreaks; these sources are indicated in the reference lists in the Technical Appendix. In addition, information about isolated outbreaks maintained by a national health system was obtained for some countries through personal contacts (see Acknowledgments). For reports in which English versions were not available, translations were obtained through the generous help of colleagues (see Acknowledgments).

## Definitions

Reports, published or unpublished, were excluded from the database if the diagnosis of *Trichinella* spp. infection was not based on a diagnostic procedure that we defined as confirmatory. Although direct demonstration of muscle larvae infection in biopsy samples is now infrequent, reliance solely on serologic testing to confirm infection can be problematic (*8*). Therefore, a serologically positive case was included in the database only if the sample was confirmed by a Western blot test or if the patient’s illness could be classified as highly probable according to the clinical diagnostic algorithm published by Dupouy-Camet and Bruschi (*7*), in which a patient with a positive serologic test result must also exhibit >1 classical trichinellosis signs and symptoms (e.g., myalgia, facial edema, headaches, diarrhea, eosinophilia).

We took a conservative approach in extracting clinical data for the analysis of frequency of major signs and symptoms in patients with *Trichinella* spp. infections and excluded report data if there was lack of clarity and reliability of clinical procedures and laboratory tests used to confirm infection. However, in some instances of inadequate clinical descriptions, the report was retained if it otherwise presented useful epidemiologic data. Consequently, the total number of human infections (Table 1, Table 2, Table 3) exceeds the number of cases used to summarize the frequency of major signs and symptoms (Table 4). Except in rare cases, clinical data were extracted from outbreak reports only if the data were from multiple cases that met these criteria; exceptions were reports of single cases from countries with rare occurrences of trichinellosis but that had good clinical and laboratory confirmation data (e.g., Korea, Japan, India).

**Table 1:** Clinically confirmed cases of trichinellosis in humans documented in World Health Organization regions, 1986–2009

Region (no. countries)	No. (%) countries with trichinellosis	No. (%) documented human infections	No. (%) deaths
African Region (46)	1 (2.17)	28 (0.04)	1 (3.57)
Region of the Americas (12)	5 (42.67)	7,179 (10.90)	10 (0.10)
Eastern Mediterranean Region (22)	2 (9.09)	50 (0.07)	0
European Region (50)	29 (58.00)	56,912 (86.47)	24 (0.04)
South-East Asian Region (11)	1 (9.69)	219 (0.33)	1 (0.50)
Western Pacific Region (27)	3 (11.11)	1,344 (2.04)	6 (0.40)
Other*	NA	86 (0.13)	0
Total (168)	41 (24)	65,818 (100.00)	42 (0.40)

*Infections acquired in countries other than the one in which diagnosis occurred. NA, not applicable

**Table 2:** Total cases and incidence of *Trichinella* spp. infections, by World Health Organization region and country, 1986–2009*

Region/country	Years	No. cases	Average incidence†
African Region, Ethiopia	1986	8	0.02
	1990	20	0.04
Region of the Americas		7,179	
Argentina	1990–2005	5,221	1.48
Canada	1987–2009	257	0.03
Chile	1991–2004	698	0.36
Mexico	1986–2001	351	0.02
United States	1987–2007	652	0.016–0.004
Eastern Mediterranean Region		50	
Iran	2007	6	0.008
Lebanon	1995	44	1.25
European Region		56,912	
Belarus	1988, 1989	16	0.08, 0.55
Bosnia and Herzegovina	1993–2003	1,600	0.1–8.0
Bulgaria	1990–2006	4,108	2.9
Croatia	1994–2009	2,110	0.02–12.3
Czech Republic	1986–2009	31	0.01
Estonia	1986–2009	91	0.0–2.9
France	1986–2009	1,203	0.00–0.95
Georgia	1988	3	0.05
Germany	1986–2009	185	0.00–0.01
Greece	2009	1	0.008
Hungary	1986–2009	158	0.18–0.057
Ireland	2007	2	0.04
Israel	2002, 2004	230	0.5, 3.0
Italy	1986–2009	1,181	0.0–0.9
Kyrgyzstan	1996	10	0.2
Latvia	1986–2009	636	0.07–3.8
Lithuania	1989–2009	3,979	0.4–21.8
Macedonia	1992	6	0.3
Poland	1986–2007	3,084	0.05–1.5
Romania	1986–2007	28,564	1.7–16.1
Russia	1996–2002	971	0.3–0.6
Serbia	1994–2003	5,210	1.8–7.8
Slovakia	1986–2008	440	0.0–6.2
Slovenia	1989–2006	203	0.00–10.5
Spain	1986–2009	1,244	0.0–0.4
Switzerland	1994, 2009	4	0.01, 0.04
Turkey	2003, 2004	425	0.01, 0.59
United Kingdom	1999	7	0.01
Ukraine	1986–2009	1,210	0.00–0.30
South-East Asian Region		219	
India	1996–2002	3	0.0003
Thailand	1993–2007	216	0.35
Western Pacific Region		1,344	
Japan	1999–2005	4	NA
South Korea	1999–2003	8	0.016
Laos	2004–2006	123	2.09
People’s Republic of China	1995–2009	1,137	NA
Singapore	1998	25	0.64
Vietnam	2001–2004	47	0.058

*The detailed data and references for each country are available in the Technical Appendix, section A. NA, insufficient data for incidence calculation. †Incidence/100,000 person-years. For some countries, incidence was not reported and was calculated from data available in the report referenced.

**Table 3:** Trichinellosis acquired in locations different from those where the disease developed and was diagnosed, 1986–2009*

Country where infection developed and was diagnosed	Country where infection was acquired (no. clinical cases)
Austria	Yugoslavia (10)
Belgium	Canada (1)
Czech Republic	Poland (2), Ukraine (2), France (1)
Denmark	Poland (12)
France	Algeria (6), Cameroon (1), Canada (13), Croatia (1), Greenland (2), Kenya (2), Laos (5), Senegal (5), Spain (1), Thailand (1), Turkey (3), Yugoslavia (1)
Germany	Canada (1), Poland (3)
Italy	Romania (4)
The Netherlands	Yugoslavia (3), Montenegro (5)
Hong Kong	Canada (1)

*Complete data and references are available in the Technical Appendix, section B.

**Table 4:** Frequency of major clinical signs associated with trichinellosis among World Health Organization regions, 1986–2009*

Region	Total no. cases†	Clinical sign, no. cases						Deaths
		Diarrhea	Myalgia	Fever	Facial and/or eyelid edema	Headache	Eosinophilia	
African Region	28	28	8	11	8	6	6	0
Region of the Americas	1,229	400	969	821	790	410	606	10
Eastern Mediterranean Region	45	43	42	41	Not reported	30	0	4
European Region	3,118	798	1,971	1,387	1,617	351	1,850	24
South-East Asian Region	210	82	206	103	102	71	97	1
Western Pacific Region	747	79	409	474	429	104	180	8
Total no. (%)	5,377 (100.0)	1,430 (27.0)	3,605 (67.0)	2,837 (53.0)	2,946 (55.0)	972 (18.0)	2,739 (51.0)	35 (1.0)

*Report references are available in the Technical Appendix, section C. †Cases included in this table were selected from all reports on the basis of detailed descriptions of clinical data in the reports.

From the original 494 abstracts identified from literature searches, 378 were judged to potentially meet the criteria for data extraction, and full articles were obtained for most abstracts. From these, 261 reports were retained for data extraction and inclusion. A major reason for rejection of articles was failure to meet the criteria for confirmation of infection, especially interpretation of serologic results.

## Incidence

In Table 2, the incidence (100,000 person-years) is reported for specific periods because the data were obtained over a shorter period than the formal study interval (1986–2009). For some countries, when incidence figures were not reported in published papers or national health reports, we calculated incidence from data available in the referenced reports by using the WHO World Population Prospects (the 2008 revision) (http://esa.un.org/wpp/unpp/p2k0data.asp). Overall, from 1986 through 2009, there were 65,818 cases and 42 deaths from trichinellosis reported from 41 countries (Tables 1–3). The European Region accounted for 86% of cases (56,912), of which 28,564 (50%) occurred in Romania, mainly during 1990–1999. The full references for specific country reports summarized in Tables 2–5 are available in the Technical Appendix.

**Table 5:** Demographic data on trichinellosis patients, by World Health Organization region and country, 1986–2009

Region/country	% Male patients (total no. cases)†	Age, y, of infected persons (no. cases)
African Region: Ethiopia	100 (28)	Range 23–25 (3); mean 24 (3)
Region of the Americas		
Canada	62.1 (150)	Range 21–67 (85); mean 34.4 (65)
Chile	60 (667)	Range 5–70 (667)
Mexico	35 (59)	Range 25–44 (59)
United States	57.5 (632)	Range 1–87 (412); mean 42.0 (126); median 37.1 (232)
Eastern Mediterranean Region: Lebanon	54 (44)	Range 10–70 (44); mean 33 (44)
European Region		
Bulgaria	49 (228)	Range 1–70 (228)
Croatia	57 (200)	Range 3–67 (200); mean 35 (200)
Czech Republic	41.9 (31)	Range 9–68 (31); mean 35.9 (31)
France	51.4 (586)	Range 1–84 (581); mean 43.8 (581)
Germany	51.9 (104)	Range 1–73 (101); mean 34.8 (101)
Israel	100 (26)	Mean 32 (26)
Italy	50.3 (382)	Range 1–90 (368); mean 36.7 (368)
Romania	53.2 (521)	Range 1- >60 (521); mean 31.4 (521)
Slovakia	63.6 (11)	Range 16–80 (21); mean 40.5 (21)
Spain	57.5 (237)	Range 2–86 (140); mean 40.7 (177)
Turkey	52.6 (418)	Range 1.5–73 (418); mean 31.1 (418)
South-East Asian Region: Thailand	71 (165)	Range 7–70 (210); mean 35.6 (208); median 34.5 (140)
Western Pacific Region		
Laos	47 (111)	Range 5–69 (111); mean 30.4 (90); median 34 (21)
People’s Republic of China	58.2 (802)	Range 1–90 (482)
Vietnam	92 (42)	Range 20–60 (42); mean 45.4 (42)
Singapore	56 (25)	Mean 22.5 (25)

*Clinical details and report references are available in the Technical Appendix, section D. †Data are from reports that presented adequate sex and age data on >10 cases during 1986–2009.

Of 46 countries in the African Region, trichinellosis has been documented only among soldiers in the Gojjam region and policemen in the Arsi region of Ethiopia, a country where the Christian population accounts for ≈60% of the total population. In the Eastern Mediterranean Region, trichinellosis was documented only in the Christian population of Lebanon and in Iran from the consumption of wild boar meat (Table 2). In Algeria and Senegal, where most of the population is Muslim, trichinellosis has been documented only in Europeans (*6*).

In the European Region, 4 epidemiologic patterns are discernable: 1) countries of eastern Europe where incidence rates are >1 case/100,000 inhabitants (Bosnia-Herzegovina, 4.1; Bulgaria, 2.4–2.9; Croatia, 1.7–4.8; Latvia, 1.1–1.3; Lithuania, 1.2–6.6; Romania, 2.9–8.5; and Serbia, 5.0); 2) countries with a low number of inhabitants where the occurrence of a large outbreak results in a high incidence rate (e.g., Israel, 3.0; Slovakia, 6.2; and Slovenia, 10.5); 3) 19 countries with a low incidence rate caused either by sporadic infections or by a large general population that reduces the incidence per 100,000 inhabitants even when a large outbreak occurs; and 4) 21 countries where no autochthonous infections were reported during the period. Incidence in eastern Europe spiked during the late 1980s and early 1990s and then decreased over the past decade. This pattern may be linked to the political, social, and economic changes that occurred with the breakup of the former Soviet Union as described by Djordjevic et al. (*9*). The gradual restoration of infrastructure related to food safety (e.g., meat inspection, pig production management, veterinary services) probably contributed substantially to the decrease in incidence in these countries.

The number of cases in the Region of the Americas was comparatively low (Table 1), except in Argentina (Table 2). National incidence estimates are limited for Region of the Americas countries and published only for the United States, Chile, and Argentina; data from Canada, Mexico, and Argentina pertain only to selected states, provinces, or districts that had large outbreaks. In Canada during the period, a few large outbreaks in northern communities among native people who consumed wild game accounted for most of the outbreaks in the country. As an example of the problem of informal or clandestine meat transportation, 2 outbreaks occurred among foreign hunters; in 1 outbreak, the hunters transported infected bear home (France) and unknowingly exposed friends and family (17 total cases). For Canada and Greenland, trichinellosis was caused by consumption of wild game harboring *T. nativa*, which does not infect swine; no pork-transmitted *Trichinella* spp. have been recorded in Canada for many years.

The risk for trichinellosis has decreased markedly in the United States and Chile since the 1990s. The large number of cases associated with Argentina contrasts with the situation among other countries in South America. The cases in Argentina may be related to the European origins of persons immigrating there and the risky food behavior they brought with them (*10*). Although incidence data from Mexico and Argentina are limited, trichinellosis outbreaks are reported frequently in Argentina from domestic pork, indicating that a substantial pig husbandry risk persists in that country.

The Asian countries of the Western Pacific Region and the South-East Asian Region reported few outbreaks during the period (Tables 1, 2). Although large outbreaks in the People’s Republic of China have been reported (*11*), the criteria for selection of reports and data extraction eliminated some reports because of insufficient diagnostic detail to meet the confirmation criteria. Most of the outbreaks reported from Thailand, Laos, and Vietnam, occurred in the northern mountainous regions among the indigenous people who practice free-roaming pig husbandry (*12**–**14*). After a 30-year period of no reports of trichinellosis cases, Laos recently experienced several outbreaks (*12*). The estimated incidence in rural areas of that country is high, which suggests a possible emerging problem there.

Globally, reporting of trichinellosis varies greatly. A major factor affecting the collection of epidemiologic and clinical data is an absent or inadequate national reporting system. For example, in some countries of eastern Europe (e.g., Bosnia-Herzegovina, Byelorussia, Georgia, Moldavia, Romania, Russia, Ukraine) trichinellosis occurs frequently in villages during the winter, and infection might not be diagnosed and subsequently reported unless infection is sufficiently severe to require hospitalization or the cases are part of a larger outbreak that requires attention from public health authorities (A. Marinculic, M.C. Cretu, W. Kociecka, N. Iashvili, N. Bogatko, pers. comm.). For example, in Romania, most of the 20,059 cases documented during 1990–1999 pertain to hospitalized persons only. However, for each hospitalized person, there are probably others in whom a moderate or mild infection developed that did not justify the travel and costs that would be incurred in seeking attention for diagnosis and treatment. Consequently, they are not usually officially recorded as having trichinellosis (M.C. Cretu, pers. comm.). In countries where most of the population is Muslim, *Trichinella* spp. infection is rare and may not be reported at all because of a scarcity of physicians, lack of good diagnostic tools, and occurrence in remote areas. In contrast, in industrialized countries such as those of Western Europe, United States, and Canada, nearly all cases are more likely to be detected and recorded, including asymptomatic cases associated with large outbreaks. For these reasons, the data we present may underrepresent the incidence of trichinellosis in lesser developed countries in comparison to that in industrialized and affluent countries.

## Sex- and Age-specific Infection

Data from clinical reports (Table 5) demonstrate that trichinellosis is a disease primarily of adults, occurring about equally among both sexes (2,631 [51%] of 5,154 infections occurred in male patients). Infection in male patients did occur more frequently, however, in Ethiopia (100%), Vietnam (91%), Japan and South Korea (75%), Thailand (64%), and China (57%). Age-specific infection data (Table 5) show the highest proportion of cases, for both sexes, was among persons 20–50 years of age (median 33.1 years). Data on age-specific prevalence rates were rarely reported; however, recent improvements in diagnosis of trichinellosis, particularly immunodiagnostic methods, may encourage more human prevalence surveys and surveillance for trichinellosis that could yield better information about sex- and age-specific rates.

Although infections also occur in children and teenagers, the predominance of infection in adults probably results from culture-driven food behavior. Improperly cooked or prepared meat dishes may be more commonly eaten at adult-oriented events, particularly if alcohol is consumed. There are only a few published studies on the link between food behavior and trichinellosis (*15**,**16*), but this potential behavioral risk factor is similar to that that occurs in other foodborne parasites, such as fish-borne parasites (*17*).

## Clinical Signs and Sequelae

For 5,377 cases, the chief clinical signs of trichinellosis were compatible in type and frequency with the classical trichinellosis syndrome (*7*), i.e., myalgia, diarrhea, fever, facial edema, and headaches that, after treatment, disappeared within 2–8 weeks (Table 4). Their rapid recovery reflects improvements in diagnostic methods, drug therapy, and public health education. The more rapid diagnosis and treatment in recent decades may also account for the low death rate; 42 deaths occurred worldwide during the 24-year period. Determining the disease burden of trichinellosis, however, is hampered by lack of data on the long-term sequelae of infection; few clinical reports included posttreatment follow-up evaluations, particularly beyond 1 month. The few studies that included follow-up over a longer time span indicate that myalgia and fatigue can persist for 4 months and, in a substantial proportion of cases, for up to 2 years (*18**–**20*). There is a need for internationally recognized epidemiologic and clinical protocols for trichinellosis outbreaks that include follow-up investigations that would facilitate reliable calculations of disease estimates.

## Sources of Infection

Domestic pigs and wild boars were the major sources of *Trichinella* spp. infection for humans, but in recent years new infection sources, particularly from exotic hosts, have emerged (Table 6). An example is the cause of outbreaks in France, where in addition to wild boar sources, most trichinellosis cases for the past 2 decades have resulted from consumption of raw horse meat, a strong food preference in French culture (*21*). In Italy, human infections from consumption of horse meat have also been documented in 2 areas (Emilia Romagna and Lombardy regions in northern Italy and the Apulia region in southern Italy), where the French fondness for raw horse meat was introduced centuries ago (*16*). In China and the Slovak Republic, dog meat was the source of infection in several outbreaks (*22**,**23*). Although Judaic and Muslim religions forbid the consumption of pork, in Israel, Lebanon, and Syria human outbreaks of trichinellosis have occurred after consumption of meat from wild boars among the Christian Arab population and immigrant laborers (*24**–**27*). Muslim populations are not entirely protected from acquiring trichinellosis, however, as demonstrated by a large outbreak in Turkey from the consumption of minced beef illegally mixed with pork of unknown origin (Table 2) (*28*).

**Table 6:** Types of meat linked to trichinellosis cases and outbreaks in the world, by World Health Organization region and country, 1986–2009*

Region/country	Meat source, % cases or outbreaks		
	Domestic pig	Wild game	Other
African Region: Ethiopia	0	100	0
Region of the Americas			
Argentina, Chile	100	0	0
Canada	0	100	0
United States	57	43	0
Mexico	86	0	14 (horse)
Eastern Mediterranean Region: Iran and Lebanon	0	100	0
European Region			
Belarus, Croatia, Georgia, Macedonia, Serbia, United Kingdom	100	0	0
Estonia, Turkey, Ukraine	50	50	0
France	0	65	35 (horse)
Germany	83	17	0
Greece, Israel	0	100	0
Hungary	52	48	0
Italy	38	38	24 (horse)
Lithuania	48	52	0
Poland	41	59	0
Romania	95	5	0
Slovakia	50	25	25 (dog)
Spain	60	40	0
South-East Asian Region: Thailand	50	50	0
Western Pacific Region			
People’s Republic of China	86	13	1 (dog and others)
Japan	25	75	0
South Korea	0	100	0
India, Laos, Papua New Guinea	50	50	0
Singapore, Vietnam	100	0	0

*Data for each country aggregated from our database of studies. Data are from reports cited in the Technical Appendix, section A.

The demographic movements of humans with culturally unique food practices, the illegal importation of uncontrolled meat from trichinellosis-endemic to -nonendemic countries, and the introduction of risky new food practices have resulted in cases in Denmark, Germany, Italy, Spain, Sweden, and the United Kingdom (Tables 2, 3) (*29**–**33*). Many cases of trichinellosis have occurred among international travelers who acquired *Trichinella* spp. infections while visiting or hunting in disease-endemic areas and the disease developed after they returned to their home countries (Table 3) (*34**–**38*).

## Issues Affecting the Effective Control of Trichinellosis

Human behavior is the biggest determinant in the persistence of trichinellosis in the face of increasing regulations directed at ensuring the safety of meat and the enhancement of good management practices in farming, especially in areas in which trichinellosis is highly endemic, such as the European and the Americas regions. Of particular concern is an increase in the association of wild animals with domestic pigs. For example, in the United States, the expansion of the range of feral pigs (wild boars) into major areas of pig production, including free-range systems, may increase the risk for incursion of *Trichinella* spp. into the human food chain (*39*). The increased frequency of outbreaks from eating pork from wild boars in Europe is believed to be related to the great increase in wild boar populations (*40*). As with other foodborne zoonoses, cultural traditions in food behavior and practices in the use of domestic and wild animals are not easily altered, and trichinellosis can be expected to remain a food-safety risk in many areas of the world for the foreseeable future.

## References

[1] Campbell WC. Historical introduction. In: Campbell WC, editor. *Trichinella* and trichinosis. New York: Plenum Press; 1983. p. 1–30.

[2] Alban L, Pozio E, Boes J, Boireau P, Boué F, Claes M, Towards a standardised surveillance for *Trichinella* in the European Union. Prev Vet Med. 2011;99:148–60. 10.1016/j.prevetmed.2011.02.00821377747

[3] Kapel CM. Changes in the EU legislation on *Trichinella* inspection—new challenges in the epidemiology. Vet Parasitol. 2005;132:189–94. 10.1016/j.vetpar.2005.05.05516039781

[4] Pyburn DG, Gamble HR, Wagstrom EA, Anderson LA, Miller LE. Trichinae certification in the United States pork industry. Vet Parasitol. 2005;132:179–83. 10.1016/j.vetpar.2005.05.05115993000

[5] Pozio E, Hoberg E, La Rosa G, Zarlenga DS. Molecular taxonomy, phylogeny and biogeography of nematodes belonging to the *Trichinella* genus. Infect Genet Evol. 2009;9:606–16. 10.1016/j.meegid.2009.03.00319460327

[6] Pozio E. World distribution of *Trichinella* spp. infections in animals and humans. Vet Parasitol. 2007;149:3–21. 10.1016/j.vetpar.2007.07.00217689195

[7] Dupouy-Camet J, Bruschi F. Management and diagnosis of human trichinellosis. In: Dupouy-Camet J, Murrell KD, editors. FAO/WHO/OIE guidelines for the surveillance, management, prevention and control of trichinellosis. Paris: World Organisation for Animal Health; 2007. p. 37–68.

[8] Gómez-Morales MA, Ludovisi A, Amati M, Cherchi S, Pezzotti P, Pozio E. Validation of an ELISA for the diagnosis of human trichinellosis. Clin Vaccine Immunol. 2008;15:1723–9. 10.1128/CVI.00257-0818827188PMC2583519

[9] Djordjevic M, Bacic M, Petricevic M, Cuperlovic K, Malakauskas A, Kapel CM, Social, political, and economic factors responsible for the reemergence of trichinellosis in Serbia: a case study. J Parasitol. 2003;89:226–31. 10.1645/0022-3395(2003)089[0226:SPAEFR]2.0.CO;212760633

[10] Pozio E, Murrell KD. Systematics and epidemiology of *Trichinella.* Adv Parasitol. 2006;63:367–439. 10.1016/S0065-308X(06)63005-417134656

[11] Cui J, Wang ZQ, Xu BL. The epidemiology of human trichinellosis in China during 2004–2009. Acta Trop. 2011;118:1–5. 10.1016/j.actatropica.2011.02.00521324302

[12] Barennes H, Sayasone S, Odermatt P, De Bruyne A, Hongsakhone S, Newton PN, A major trichinellosis outbreak suggesting a high endemicity of *Trichinella* infection in northern Laos. Am J Trop Med Hyg. 2008;78:40–4.18187783PMC7611085

[13] Kaewpitoon N, Kaewpitoon SJ, Pengsaa P. Food-borne parasitic zoonosis: distribution of trichinosis in Thailand. World J Gastroenterol. 2008;14:3471–5. 10.3748/wjg.14.347118567073PMC2716607

[14] Vu Thi N, Dorny P, La Rosa G, To Long T, Nguyen Van C, Pozio E. High prevalence of anti-*Trichinella* IgG in domestic pigs of the Son La province, Vietnam. Vet Parasitol. 2010;168:136–40. 10.1016/j.vetpar.2009.10.02419962244

[15] Blaga R, Durand B, Antoniu S, Gherman C, Cretu CM, Cozma V, A dramatic increase in the incidence of human trichinellosis in Romania over the past 25 years: impact of political changes and regional food habits. Am J Trop Med Hyg. 2007;76:983–6.17488927

[16] Gottstein B, Pozio E, Nöckler K. Epidemiology, diagnosis, treatment, and control of trichinellosis. Clin Microbiol Rev. 2009;22:127–45. 10.1128/CMR.00026-0819136437PMC2620635

[17] Chai JY, Murrell KD, Lymbery AJ. Fish-borne parasitic zoonoses: status and issues. Int J Parasitol. 2005;35:1233–54. 10.1016/j.ijpara.2005.07.01316143336

[18] Jongwutiwes S, Chantachum N, Kraivichian P, Siriyasatien P, Putaporntip C, Tamburrini A, First outbreak of human trichinellosis caused by *Trichinella pseudospiralis.* Clin Infect Dis. 1998;26:111–5. 10.1086/5162789455518

[19] Watt G, Saisorn S, Jongsakul K, Sakolvaree Y, Chaicumpa W. Blinded, placebo-controlled trial of antiparasitic drugs for trichinosis myositis. J Infect Dis. 2000;182:371–4. 10.1086/31564510882628

[20] Calcagno MA, Teixeira C, Forastiero MA, Costantino SN, Venturiello SM. Clinical, serological and parasitological aspects of an outbreak of human trichinellosis in Villa Mercedes, San Luis, Argentina. The acute and chronic phases of the infection [in Spanish]. Medicina (B Aires). 2005;65:302–6.16193706

[21] Boireau P, Vallée I, Roman T, Perret C, Mingyuan L, Gamble HR, *Trichinella* in horses: a low frequency infection with high human risk. Vet Parasitol. 2000;93:309–20. 10.1016/S0304-4017(00)00348-411099844

[22] Dubinský P, Stefancíková A, Kinceková J, Ondriska F, Reiterová K, Medvedová M. Trichinellosis in the Slovak Republic. Parasite. 2001;8(Suppl):S100–2.1148432610.1051/parasite/200108s2100

[23] Liu M, Boireau P. Trichinellosis in China: epidemiology and control. Trends Parasitol. 2002;18:553–6. 10.1016/S1471-4922(02)02401-712482541

[24] Haim M, Efrat M, Wilson M, Schantz PM, Cohen D, Shemer J. An outbreak of *Trichinella spiralis* infection in southern Lebanon. Epidemiol Infect. 1997;119:357–62. 10.1017/S09502688970078759440440PMC2809009

[25] Hefer E, Rishpon S, Volovik I. Trichinosis outbreak among Thai immigrant workers in the Hadera sub-district [in Hebrew]. Harefuah. 2004;143:656–60, 694.15521681

[26] Marva E, Markovics A, Gdalevich M, Asor N, Sadik C, Leventhal A. Trichinellosis outbreak. Emerg Infect Dis. 2005;11:1979–81.1648550010.3201/eid1112.050461PMC3367633

[27] Olaison L, Ljungström I. An outbreak of trichinosis in Lebanon. Trans R Soc Trop Med Hyg. 1992;86:658–60. 10.1016/0035-9203(92)90178-F1287937

[28] Akkoc N, Kuruuzum Z, Akar S, Yuce A, Onen F, Yapar N, A large-scale outbreak of trichinellosis caused by *Trichinella britovi* in Turkey. Zoonoses Public Health. 2009;56:65–70. 10.1111/j.1863-2378.2008.01158.x18705658

[29] Milne LM, Bhagani S, Bannister BA, Laitner SM, Moore P, Eza D, Trichinellosis acquired in the United Kingdom. Epidemiol Infect. 2001;127:359–63. 10.1017/S095026880100599411693515PMC2869757

[30] Pozio E, Marucci G. *Trichinella*-infected pork products: a dangerous gift. Trends Parasitol. 2003;19:338. 10.1016/S1471-4922(03)00138-712901932

[31] Gallardo MT, Mateos L, Artieda J, Wesslen L, Ruiz C, García MA, Outbreak of trichinellosis in Spain and Sweden due to consumption of wild boar meat contaminated with *Trichinella britovi*. Euro Surveill. 2007;12:E070315.1.10.2807/esw.12.11.03154-en17439785

[32] Nöckler K, Wichmann-Schauer H, Hiller P, Müller A, Bogner K. Trichinellosis outbreak in Bavaria caused by cured sausage from Romania, January 2007. Euro Surveill. 2007;12:E070823.2.10.2807/esw.12.34.03254-en17868621

[33] Stensvold CR, Nielsen HV, Mølbak K. A case of trichinellosis in Denmark, imported from Poland, June 2007. Euro Surveill. 2007;12:E070809.3.10.2807/esw.12.32.03248-en17868552

[34] Dupouy-Camet J, Allegretti S, Truong TP. Enquete sur l’incidence de la trichinellose en France (1994–1995). Bulletin Épidémiologique Hebdomadaire. 1998;28:122–3.

[35] McAuley JB, Michelson MK, Schantz PM. Trichinellosis surveillance, United States, 1987–1990. MMWR CDC Surveill Summ. 1991;40:35–42.1770927

[36] Shiota T, Arizono N, Yoshioka T, Ishikawa Y, Fujitake J, Fujii H, Imported trichinellosis with severe myositis—report of a case [in Japanese]. Kansenshogaku Zasshi. 1999;73:76–82.1007790510.11150/kansenshogakuzasshi1970.73.76

[37] Ancelle T, De Bruyne A, Niang M, Poisson D-M, Prazuck T, Fur A, Outbreak of trichinellosis caused by *Trichinella nativa* due to consumption of bear meat [in French]. Bulletin Épidémiologique Hebdomadaire. 2006;14:96–8.

[38] Houzé S, Ancelle T, Matra R, Boceno C, Carlier Y, Gajadhar AA, Trichinellosis acquired in Nunavut, Canada in September 2009: meat from grizzly bear suspected. Euro Surveill. 2009;14:pii=19383.19941776

[39] Burke R, Masuoka P, Murrell KD. Swine *Trichinella* infection and geographic information system tools. Emerg Infect Dis. 2008;14:1109–11. 10.3201/eid14.07.07153818598633PMC2600339

[40] Sáaez-Royuela C, Telleriia JL. The increased population of the wild boar (*Sus scrofa* L.) in Europe. Mammal Rev. 1986;16:97–101. 10.1111/j.1365-2907.1986.tb00027.x

# Review article: Worldwide Occurrence and Impact of Human Trichinellosis, 1986–2009

**Authors:** K. Darwin Murrell, Edoardo Pozio

**Affiliations:** Author affiliations: University of Copenhagen, Copenhagen, Denmark (K.D. Murrell);; Istituto Superiore di Sanità, Rome, Italy (E. Pozio)

## Earning MEDSCAPE CME Credit

To obtain credit, you should first read the journal article. After reading the article, you should be able to answer the following, related, multiple-choice questions. To complete the questions (with a minimum 70% passing score) and earn continuing medical education (CME) credit, please go to www.medscape.org/journal/eid. Credit cannot be obtained for tests completed on paper, although you may use the worksheet below to keep a record of your answers. You must be a registered user on Medscape.org. If you are not registered on Medscape.org, please click on the New Users: Free Registration link on the left hand side of the website to register. Only one answer is correct for each question. Once you successfully answer all post-test questions you will be able to view and/or print your certificate. For questions regarding the content of this activity, contact the accredited provider, CME@medscape.net. For technical assistance, contact CME@webmd.net. American Medical Association’s Physician’s Recognition Award (AMA PRA) credits are accepted in the US as evidence of participation in CME activities. For further information on this award, please refer to http://www.ama-assn.org/ama/pub/category/2922.html. The AMA has determined that physicians not licensed in the US who participate in this CME activity are eligible for *AMA PRA Category 1 Credits*™. Through agreements that the AMA has made with agencies in some countries, AMA PRA credit may be acceptable as evidence of participation in CME activities. If you are not licensed in the US, please complete the questions online, print the certificate and present it to your national medical association for review.

## Article Title: Worldwide Occurrence and Impact of Human Trichinellosis, 1986–2009

## MEDSCAPE CME Questions

**1. You are seeing a 40-year-old man with a 1-month history of myalgia, diarrhea, and intermittent fever. He has a history of eating raw meat on a hunting trip 6 weeks ago.You suspect that this patient might have trichinellosis. What should you consider regarding the epidemiology of trichinellosis in the current study?**A. Trichinellosis was particularly common among MuslimsB. Western Europe had higher rates of trichinellosis compared with Eastern EuropeC. The prevalence of trichinellosis in the United States has risen dramaticallyD. Argentina has the highest prevalence of trichinellosis in South America**2. Which of the following epidemiologic trends in trichinellosis was most pronounced in the current study?**A. Children had higher rates of trichinellosis compared with adultsB. Adults had higher rates of trichinellosis compared with childrenC. Women had higher rates of trichinellosis compared with menD. Men had higher rates of trichinellosis compared with women**3. What should you consider regarding the diagnosis and treatment of trichinellosis in this patient?**A. Symptoms of myalgia, diarrhea, and fever are commonB. Symptoms usually resolve within a few days of the initiation of therapyC. No long-term sequelae to infection have been describedD. Trichinellosis is still usually fatal**4. Which of the following animals was the most common source of trichinellosis in the current study?**A. HorseB. DogC. CowD. Pig

## Activity Evaluation

**1:** The activity supported the learning objectives.

Strongly Disagree				Strongly Agree
1	2	3	4	5
**2. The material was organized clearly for learning to occur.**				
Strongly Disagree				Strongly Agree
1	2	3	4	5
**3. The content learned from this activity will impact my practice.**				
Strongly Disagree				Strongly Agree
1	2	3	4	5
**4. The activity was presented objectively and free of commercial bias.**				
Strongly Disagree				Strongly Agree
1	2	3	4	5

## Supplementary Material

Technical Appendix Additiona information on Trichinellosis infections and resources used in this study.
